# Supplementary figures and images for: Fatty acid desaturases link cell metabolism pathways to promote proliferation of Epstein-Barr virus-infected B cells
Source: PLoS Pathog. 2025 May 22;21(5):e1012685. doi: 10.1371/journal.ppat.1012685 (PMC12143519; doi:10.1371/journal.ppat.1012685)

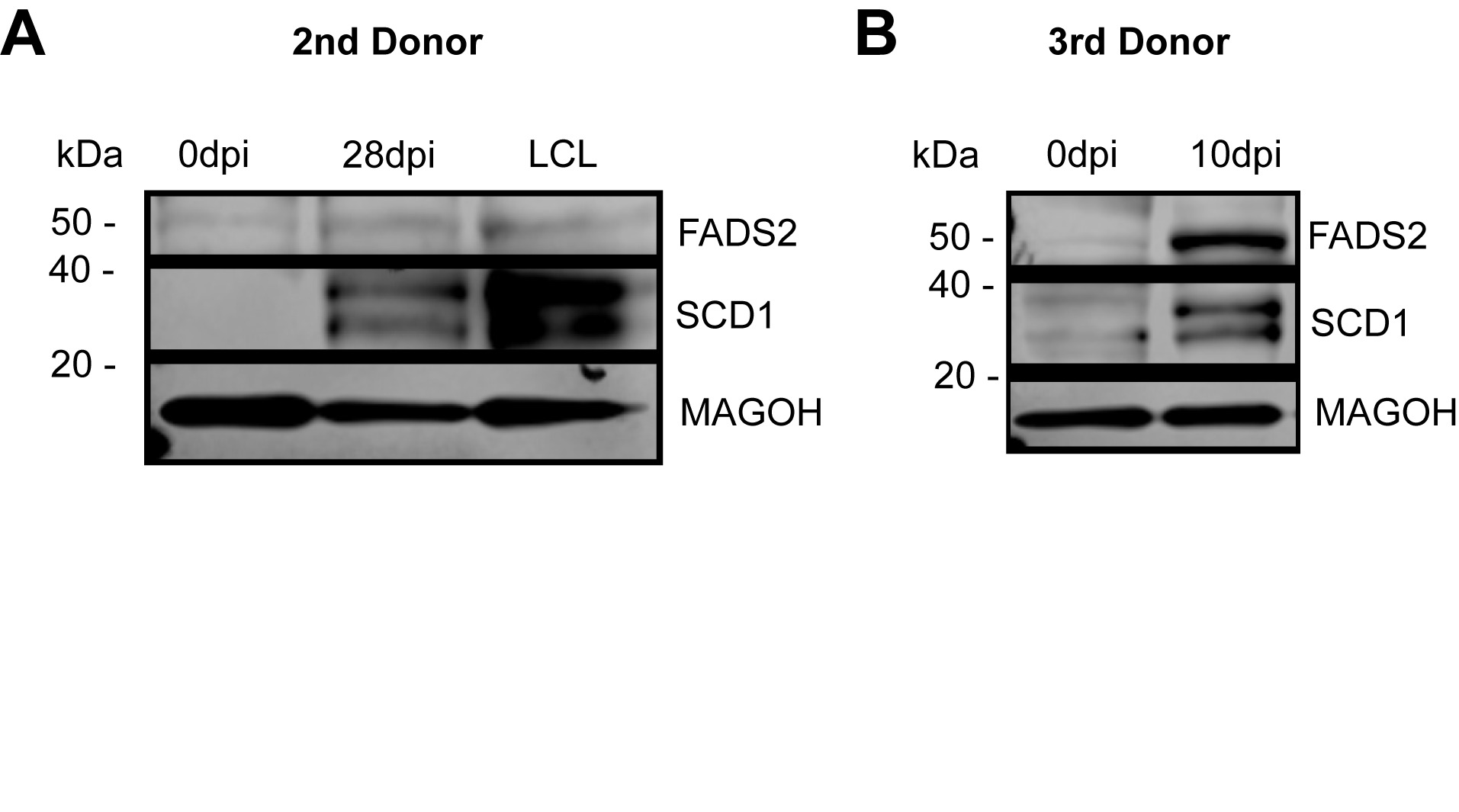

Supplement: S1 Fig — (TIF) [file ppat.1012685.s001.tif]

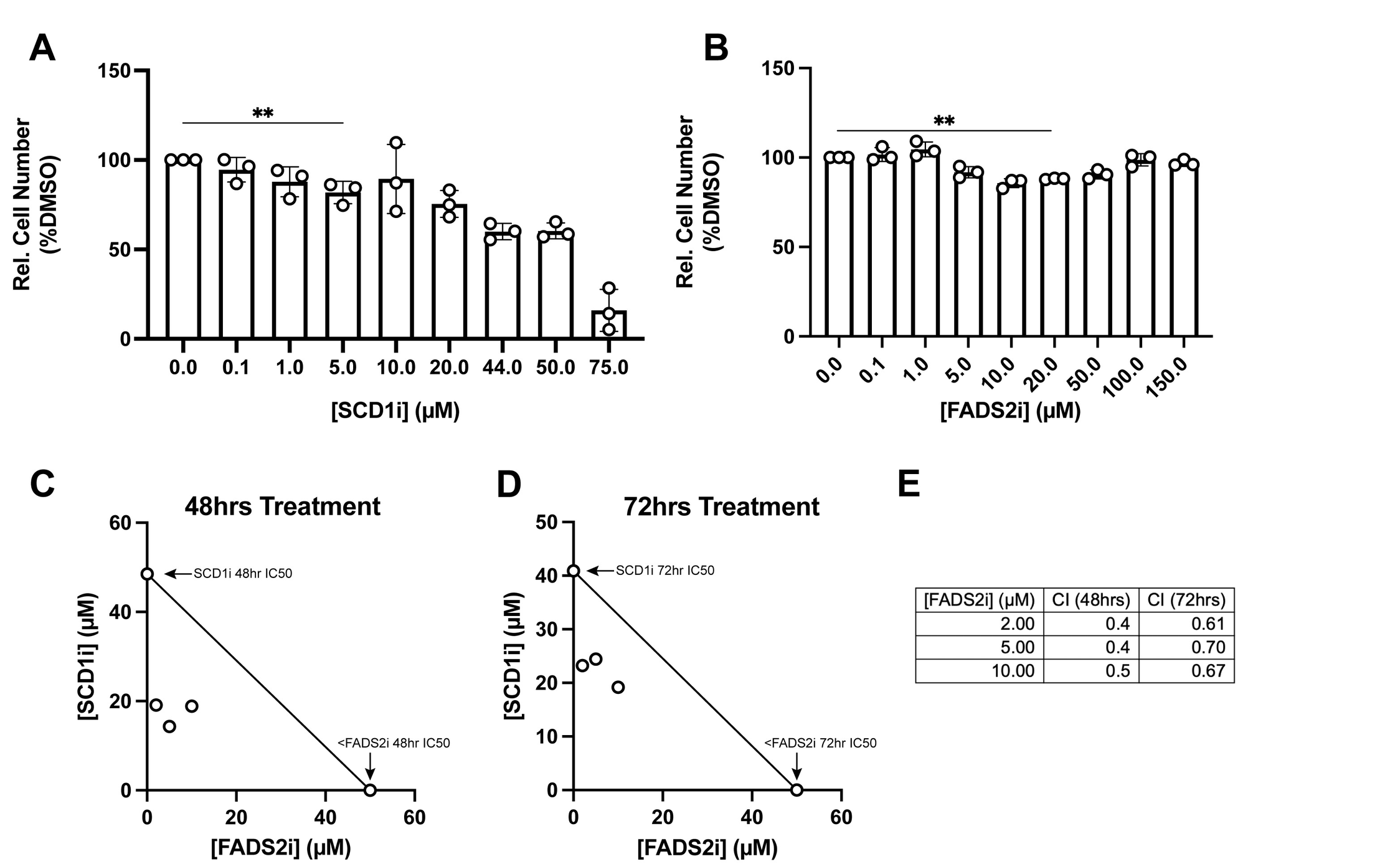

Supplement: S2 Fig — (A) Dose-response data for SCD1i. Relative cell number measured using CellTiter Glo and normalized to DMSO. (B) Dose-response data for FADS2i. Relative cell number measured using CellTiter Glo and normalized to DMSO. (C-D) Isobologram at 48 hrs and 72 hrs, respectively, using IC50 for SCD1i on y axis and sub-IC50 (maximum soluble concentration) for FADS2i on x axis. Plotted points show IC50 values for SCD1i in the presence of that concentration of FADS2i. (E) Drug combination indices (CI) at indicated dose of FADS2i. A CI value less than 1 indicates synergism. Statistical significance for pairwise comparisons determined using an unpaired Student’s two-tailed T test (* p < 0.05, **p < 0.005, ***p < 0.0005, ****p < 0.00005). (TIF) [file ppat.1012685.s002.tif]

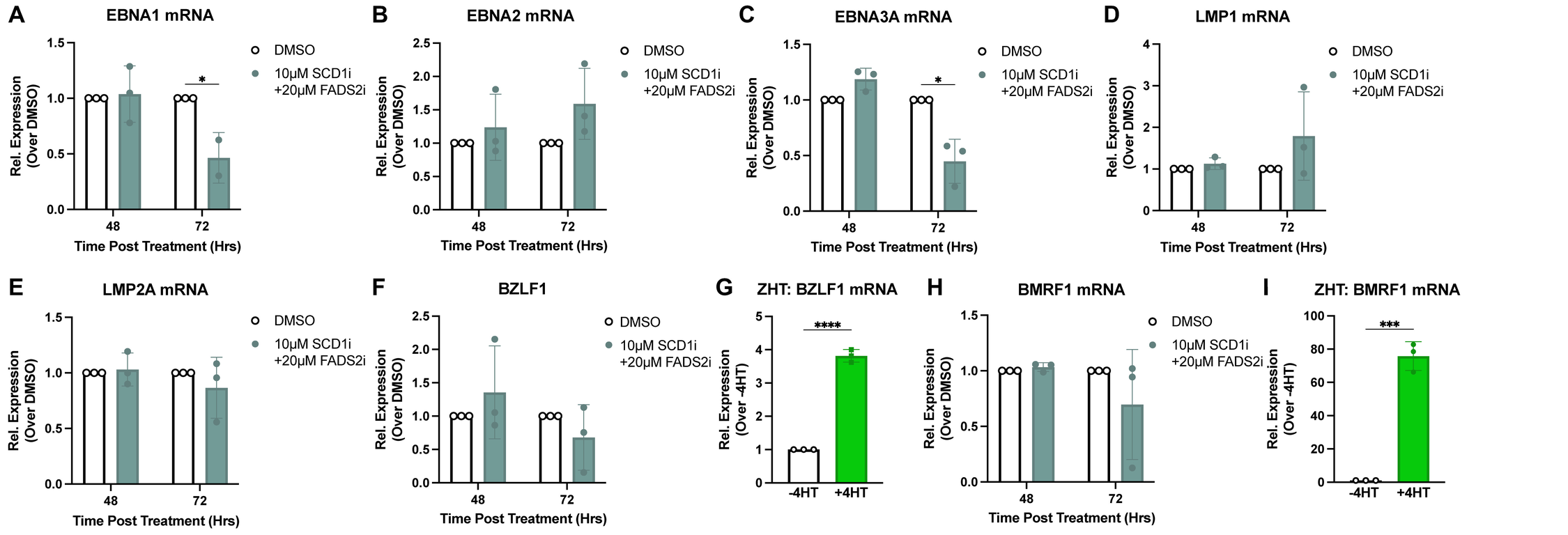

Supplement: S3 Fig — (A-E) qPCR data showing relative expression of latency-associated EBV genes, relative to DMSO. Setdb1 used as a housekeeping gene for normalization. (F-I) qPCR data showing relative expression of lytic-associated EBV genes, relative to DMSO. Setdb1 used as a housekeeping gene for normalization. The ZHT cell line treated with 4-hydroxytamoxifen (4HT) to induce lytic reactivation was included as a positive control. Statistical significance for pairwise comparisons determined using an unpaired Student’s two-tailed T test (* p < 0.05, **p < 0.005, ***p < 0.0005, ****p < 0.00005). (TIF) [file ppat.1012685.s003.tif]

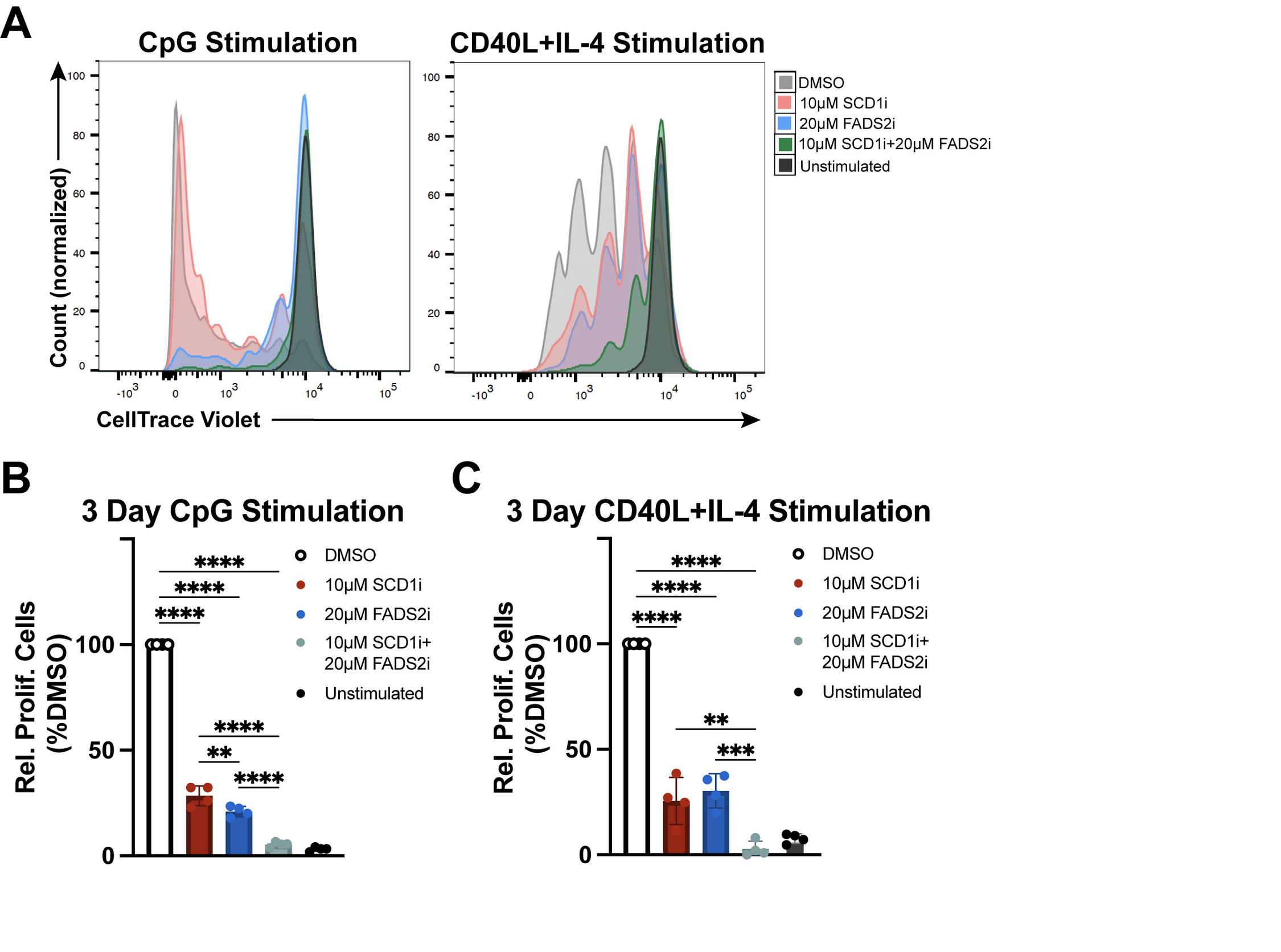

Supplement: S4 Fig — (A) Representative flow cytometry histograms five days post stimulation, showing CellTrace Violet dilution as cells divide. Counts are normalized to mode. (B) Graphs showing the number of B cells that proliferated (CD19 +/CTVlo) three days after treatment and stimulation with CpG. Cell numbers normalized to counting beads and presented as a percentage of DMSO-treated controls (C) Graphs showing the number of B cells that proliferated (CD19 +/CTVlo) three days after treatment and stimulation with recombinant CD40L and IL-4. Cell numbers normalized to counting beads and presented as a percentage of DMSO-treated controls. Statistical significance for pairwise comparisons determined using a Tukey’s post-hoc test (* p < 0.05, **p < 0.005, ***p < 0.0005, ****p < 0.00005). (TIF) [file ppat.1012685.s004.tif]

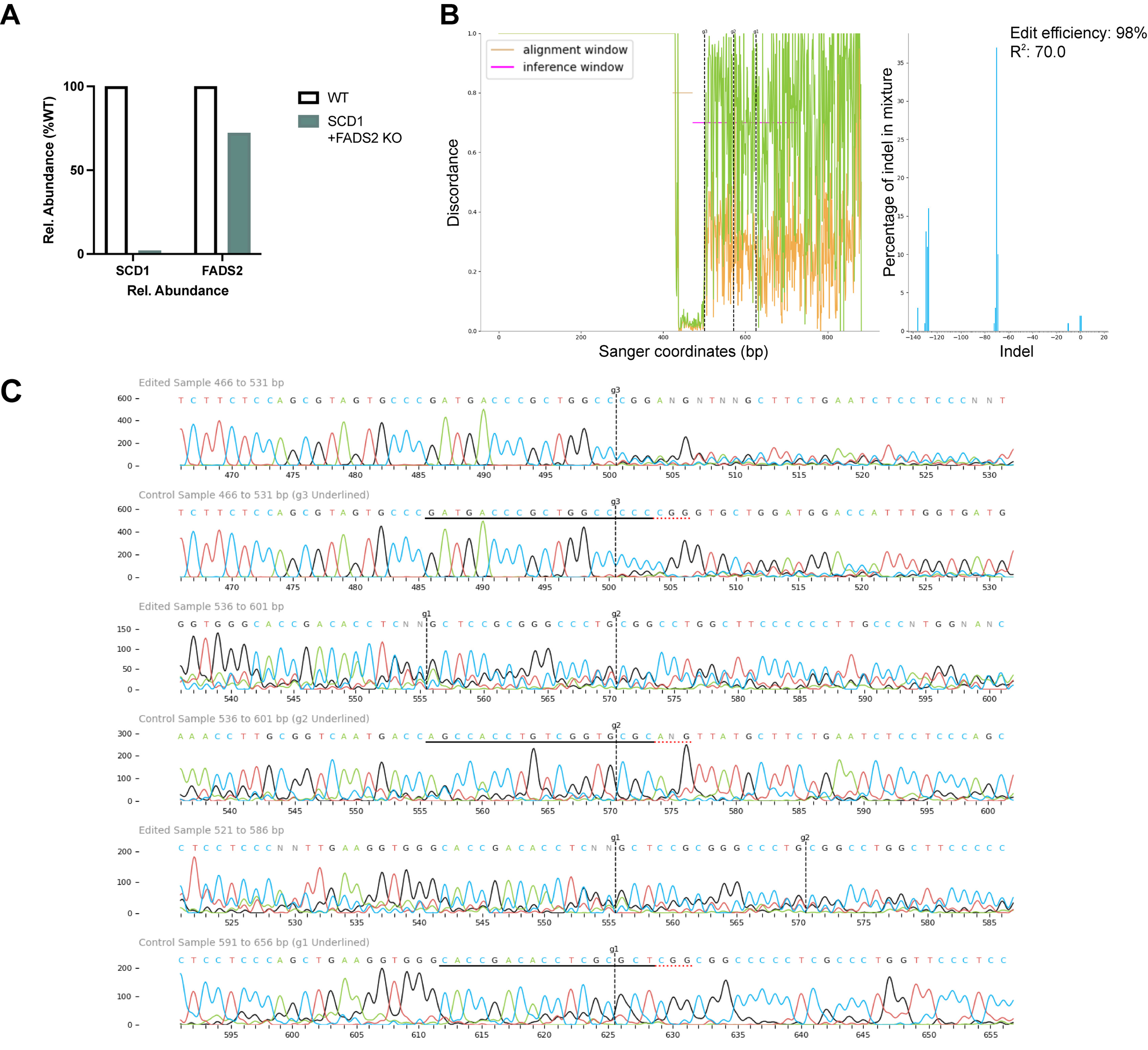

Supplement: S5 Fig — (A) Quantification of Western blot in Fig 3E, normalized to MAGOH and WT. (B) Discordance of FADS2 genomic sequences between WT and CD46 + FADS2 KO LCLs (left), and estimated percentage of each indel in mixture (right). Graphs generated using Synthego’s Inference of CRISPR Edits tool. (C) Genomic FADS2 sequences in WT and FADS2-KO LCLs. Black lines indicate binding sites of FADS2 sgRNAs. Figure generated using Synthego’s Inference of CRISPR Edits tool. (TIF) [file ppat.1012685.s005.tif]

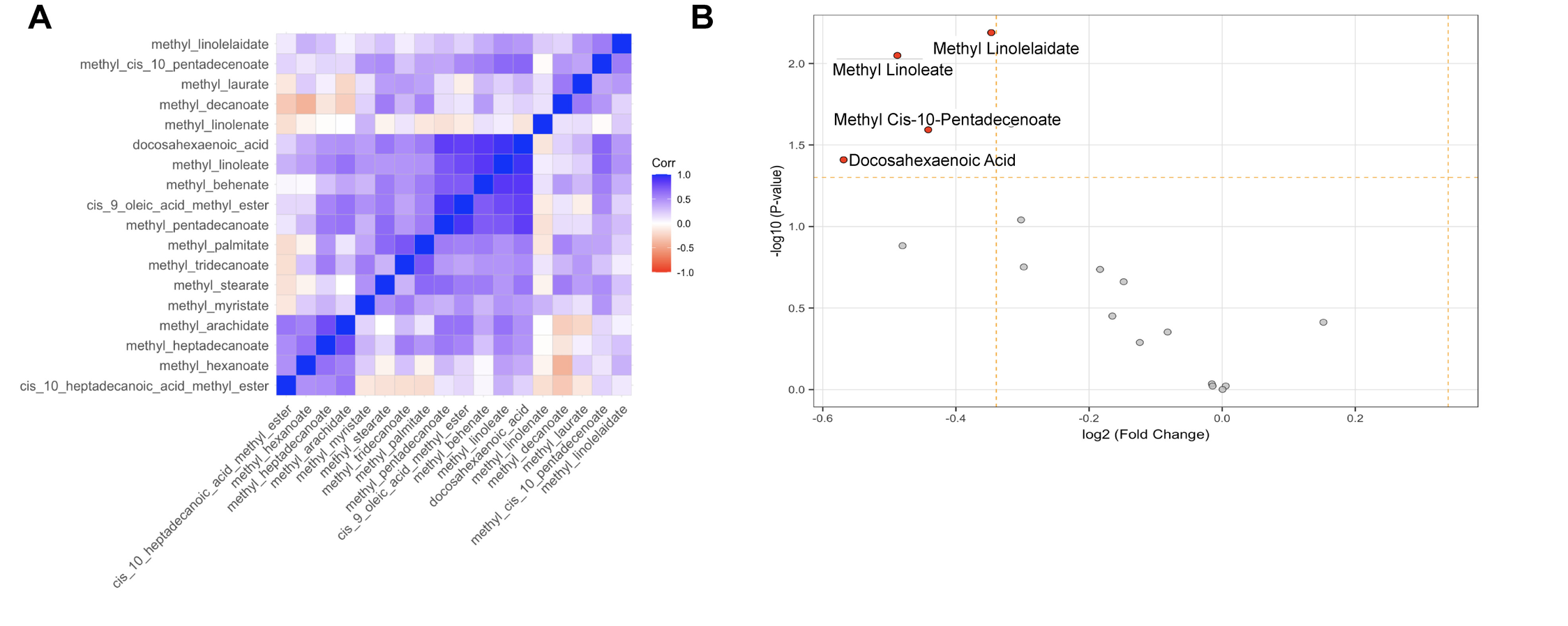

Supplement: S6 Fig — (A) Heatmap showing Pearson’s correlation coefficient values between pairs of fatty acid species. (B) Plot showing free fatty acid species with significant levels of enrichment in samples treated with 10 μM SCD1i+20 μM FADS2i. P values were determined using limma analysis of normalized FAME free fatty acid profiling data. A negative log2 fold change indicates enrichment in the SCD1i+FADS2i-treated samples, while a positive log2 fold change indicates enrichment in the DMSO-treated samples. (TIF) [file ppat.1012685.s006.tif]

Uncropped Western blot corresponding to Figure 3E

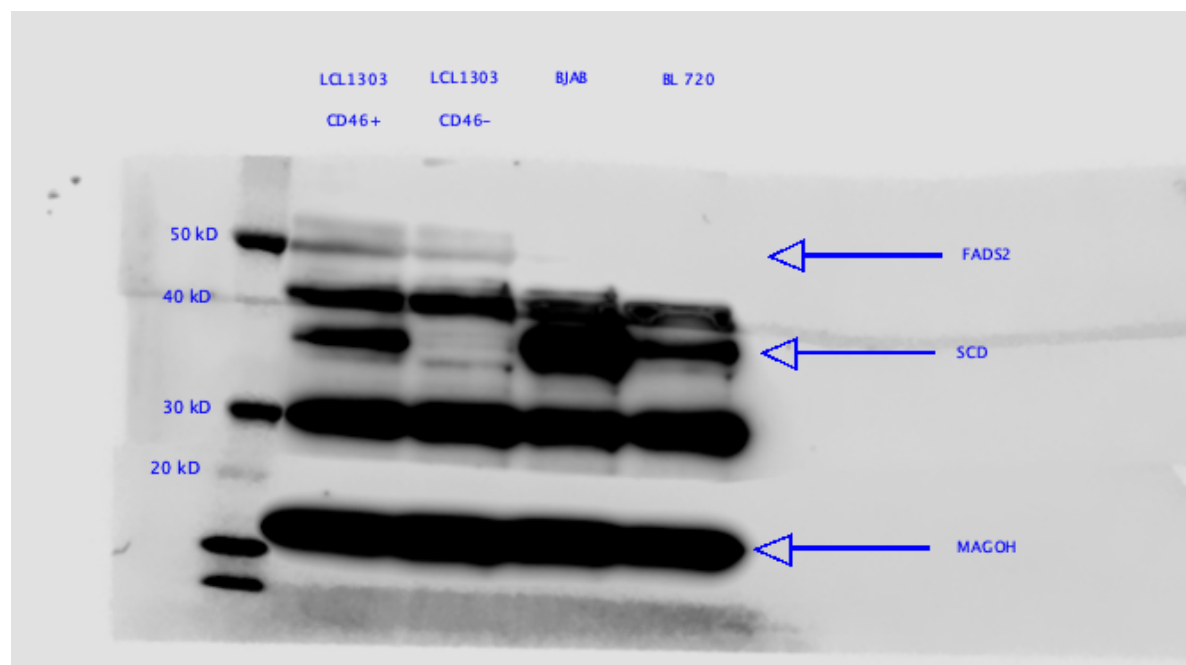

Supplement: S3 File — (A) Gating strategy corresponding to flow cytometry data displayed in Fig 3B, C. (B) Complete flow cytometry dot plots at 3 days post transfection, corresponding to Fig 3B, C. (C) Complete flow cytometry dot plots at 10 days post transfection, corresponding to Fig 3B, C. (D) Raw data corresponding to Fig 3D. (E) Uncropped Western blot corresponding to Fig 3E. (ZIP) [file ppat.1012685.s009.zip › S3_File/E_Fig3E.pdf]

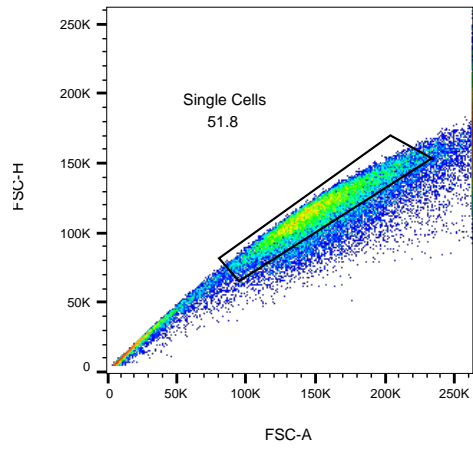

1390\_48-BSA.fcs  
 Ungated  
 32616

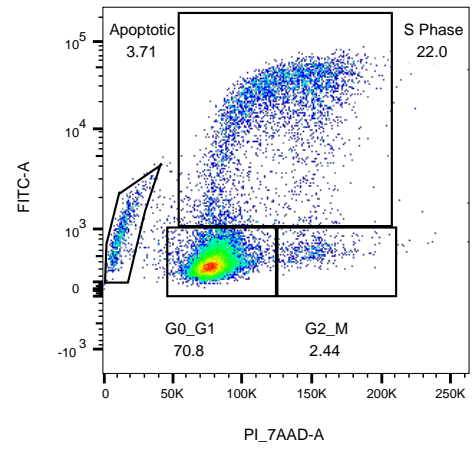

1390\_48-BSA.fcs  
 Single Cells  
 16893

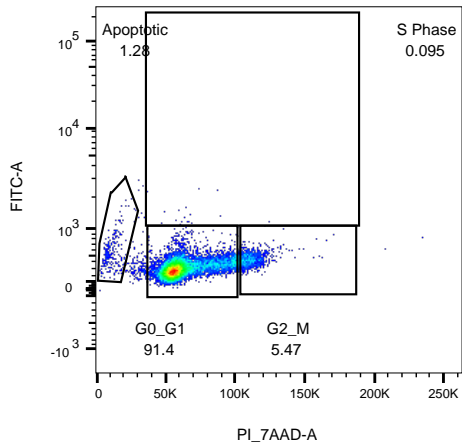

1303\_NoBrdU.fcs  
 Single Cells  
 17811

Supplement: S4 File — (A) Raw data corresponding to Fig 4B, C. (B) Gating strategy corresponding to flow cytometry data in Fig 4D, E. (C) Complete flow cytometry dot plots corresponding to Fig 4D, E. (D) Raw data corresponding to Fig 4D, E. (E) Raw data corresponding to Fig 4F. (F) Raw data corresponding to Fig 4G. (G) Gating strategy for Annexin-V measurement, corresponding to Fig 4H. (H) Gating strategy for cleaved caspase 3/7 measurement, corresponding to Fig 4H. (I) Raw data corresponding to Fig 4H. (ZIP) [file ppat.1012685.s010.zip › S4_File/B_Fig4_D_GatingStrategy.pdf]

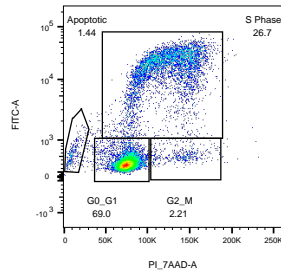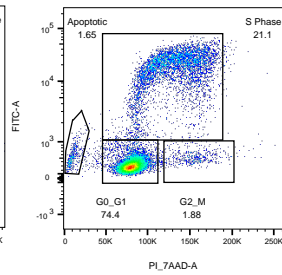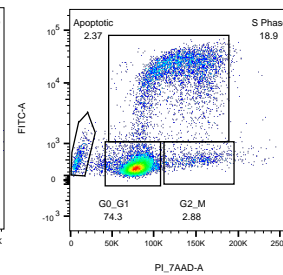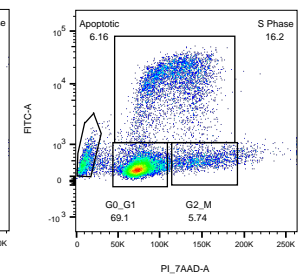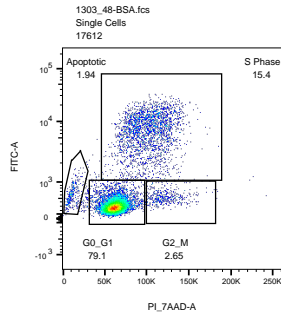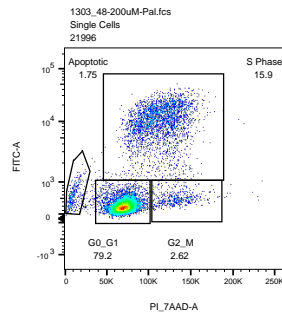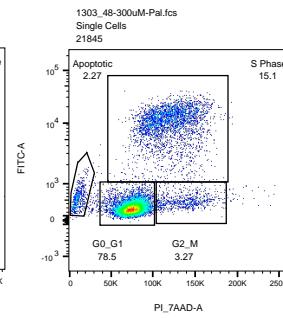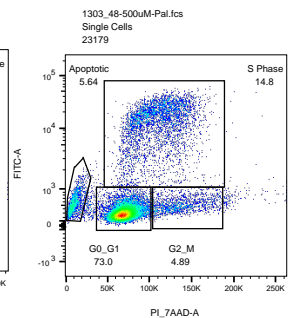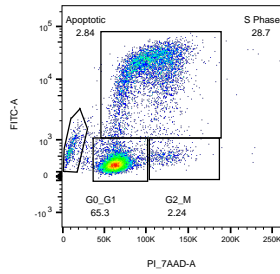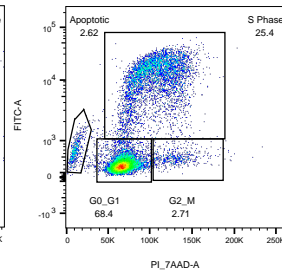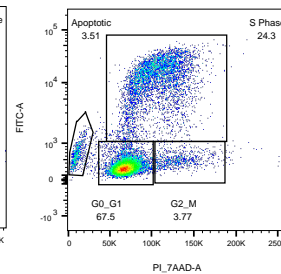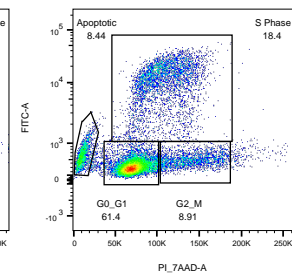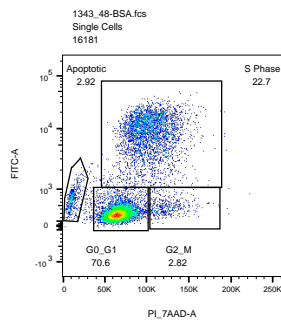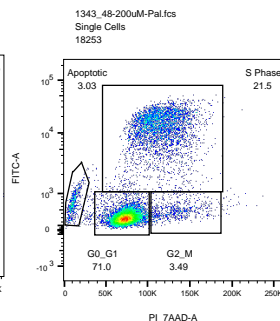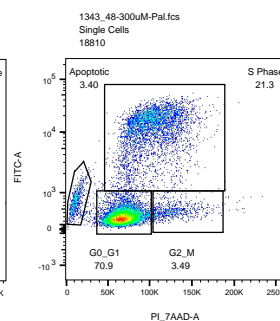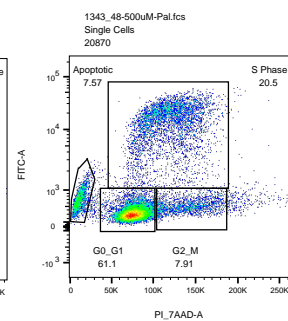

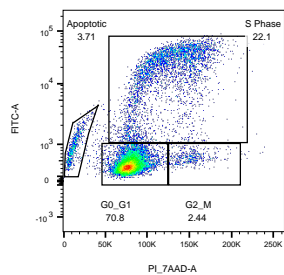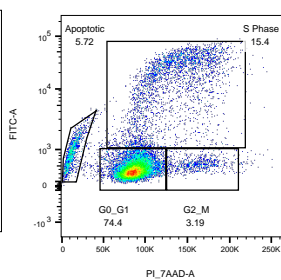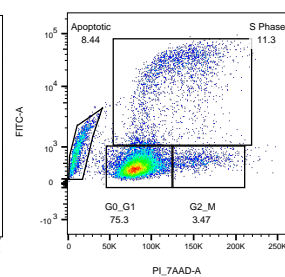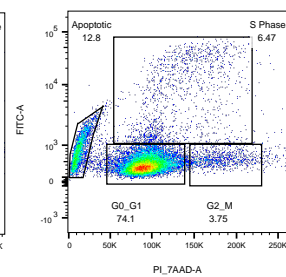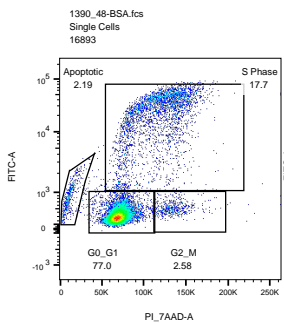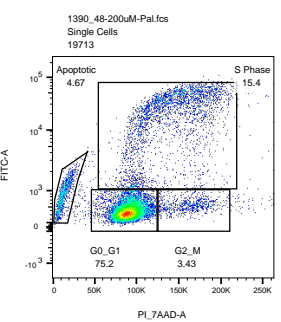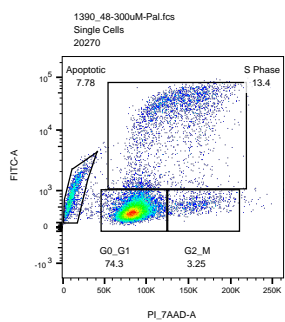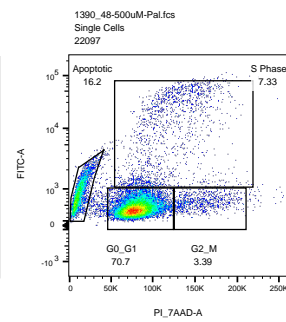

Supplement: S4 File — (A) Raw data corresponding to Fig 4B, C. (B) Gating strategy corresponding to flow cytometry data in Fig 4D, E. (C) Complete flow cytometry dot plots corresponding to Fig 4D, E. (D) Raw data corresponding to Fig 4D, E. (E) Raw data corresponding to Fig 4F. (F) Raw data corresponding to Fig 4G. (G) Gating strategy for Annexin-V measurement, corresponding to Fig 4H. (H) Gating strategy for cleaved caspase 3/7 measurement, corresponding to Fig 4H. (I) Raw data corresponding to Fig 4H. (ZIP) [file ppat.1012685.s010.zip › S4_File/C_Fig4_D.pdf]
