# Supplementary material for: Fatty acid desaturases link cell metabolism pathways to promote proliferation of Epstein-Barr virus-infected B cells
Source: PLoS Pathog. 2025 May 22;21(5):e1012685. doi: 10.1371/journal.ppat.1012685 (PMC12143519; doi:10.1371/journal.ppat.1012685)

Uncropped Western blots from Figure 1E. Only relevant lanes shown.

αFADS2

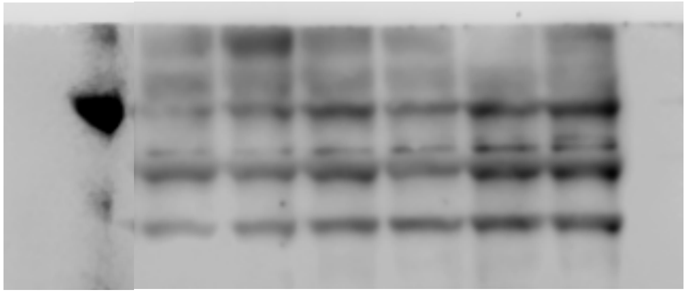

αSCD1

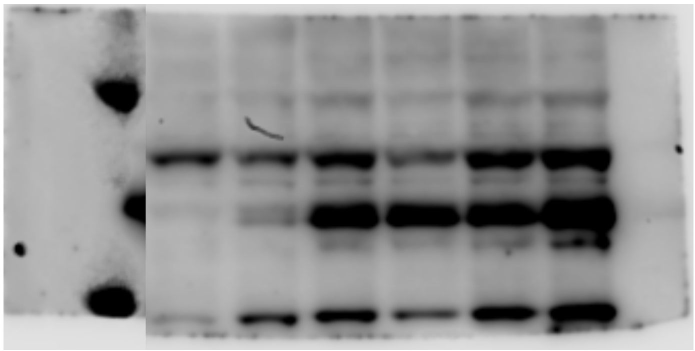

αMAGOH

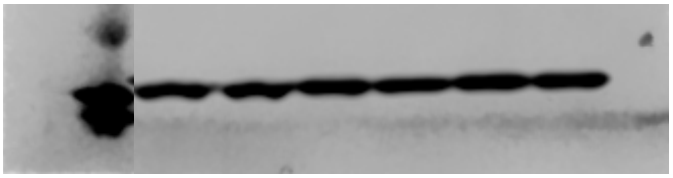

Supplement: S1 File — (A) Raw data corresponding to Fig 1C. (B) Uncropped Western blots corresponding to Fig 1E. (C) Raw data corresponding to Fig 1F. (ZIP) [file ppat.1012685.s007.zip › S1_File/B_Fig1E_Westerns.pdf]
