# Supplementary material for: Fatty acid desaturases link cell metabolism pathways to promote proliferation of Epstein-Barr virus-infected B cells
Source: PLoS Pathog. 2025 May 22;21(5):e1012685. doi: 10.1371/journal.ppat.1012685 (PMC12143519; doi:10.1371/journal.ppat.1012685)
Supplement: S3 File — (A) Gating strategy corresponding to flow cytometry data displayed in Fig 3B, C. (B) Complete flow cytometry dot plots at 3 days post transfection, corresponding to Fig 3B, C. (C) Complete flow cytometry dot plots at 10 days post transfection, corresponding to Fig 3B, C. (D) Raw data corresponding to Fig 3D. (E) Uncropped Western blot corresponding to Fig 3E. (ZIP) [file ppat.1012685.s009.zip › S3_File/A_Fig3_B-D_GatingStrategy.pdf]

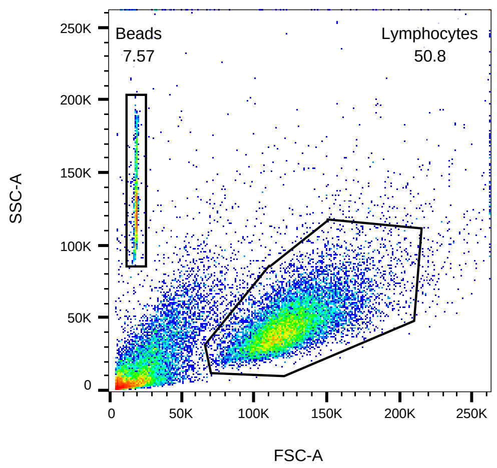

LCL1303_Stained_Untransfected_002.fcs
Ungated
26077

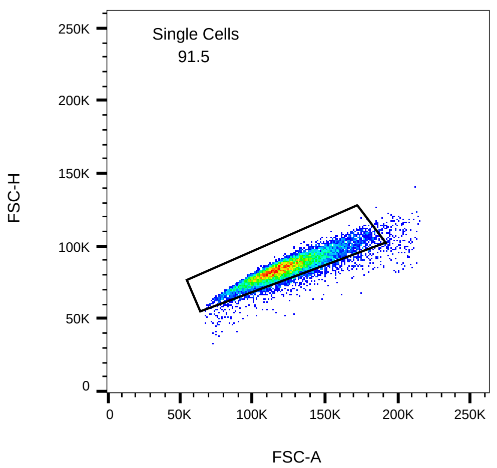

LCL1303_Stained_Untransfected_002.fcs
Lymphocytes
13244

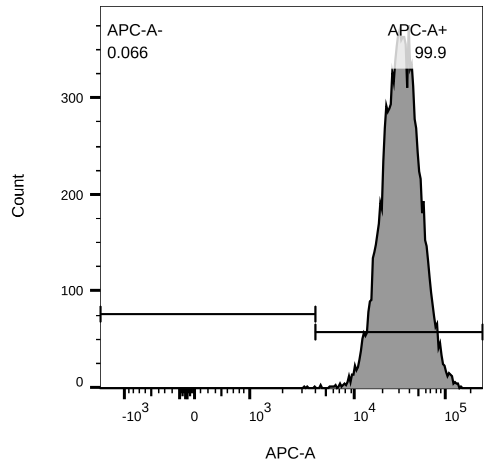

LCL1303_Stained_Untransfected_002.fcs
Single Cells
12116

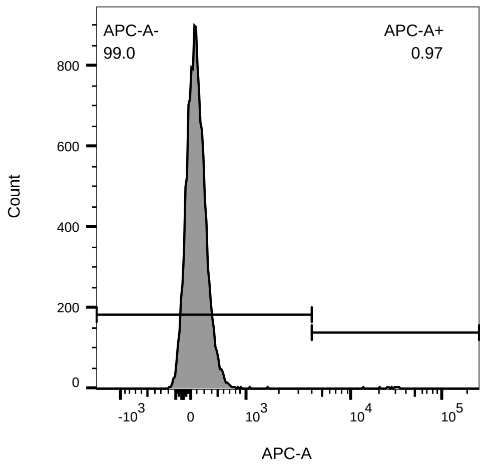

LCL1303_Unstained_Untransfected_001.fcs
Single Cells
13849

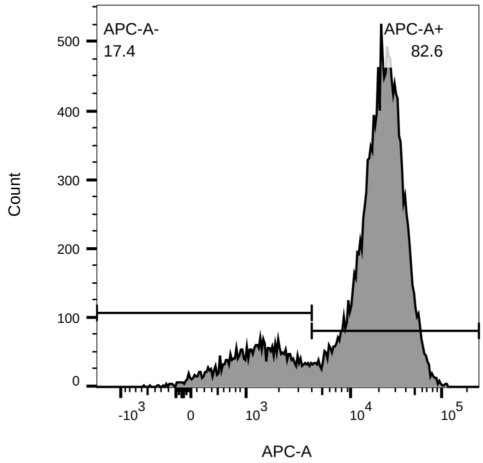

LCL1303_CD46KO-2_004.fcs
Single Cells
20700
