# Supplementary material for: Fatty acid desaturases link cell metabolism pathways to promote proliferation of Epstein-Barr virus-infected B cells
Source: PLoS Pathog. 2025 May 22;21(5):e1012685. doi: 10.1371/journal.ppat.1012685 (PMC12143519; doi:10.1371/journal.ppat.1012685)
Supplement: S3 File — (A) Gating strategy corresponding to flow cytometry data displayed in Fig 3B, C. (B) Complete flow cytometry dot plots at 3 days post transfection, corresponding to Fig 3B, C. (C) Complete flow cytometry dot plots at 10 days post transfection, corresponding to Fig 3B, C. (D) Raw data corresponding to Fig 3D. (E) Uncropped Western blot corresponding to Fig 3E. (ZIP) [file ppat.1012685.s009.zip › S3_File/B_Fig3_B-day3.pdf]

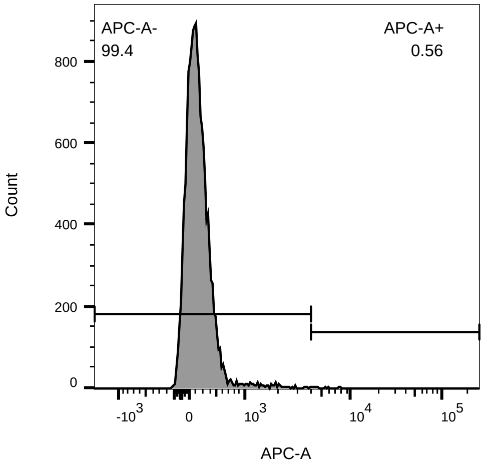

LCL472_Unstained_Untransfected_013.fcs
Single Cells
15448

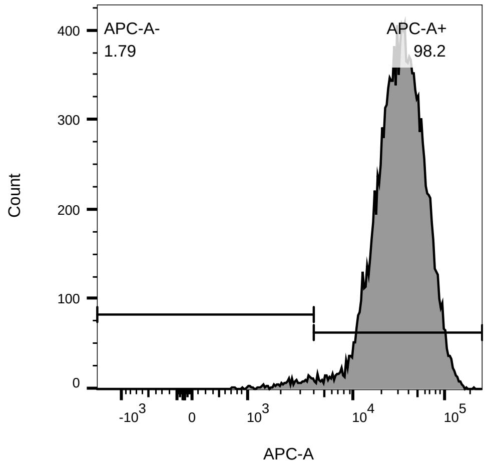

LCL472_Stained_Untransfected_014.fcs
Single Cells
16494

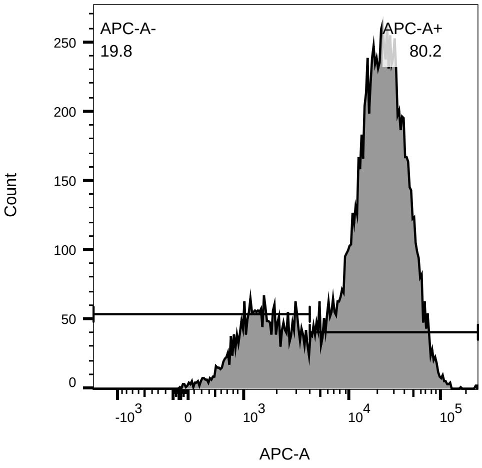

LCL472_CD46KO-2_016.fcs
Single Cells
14477

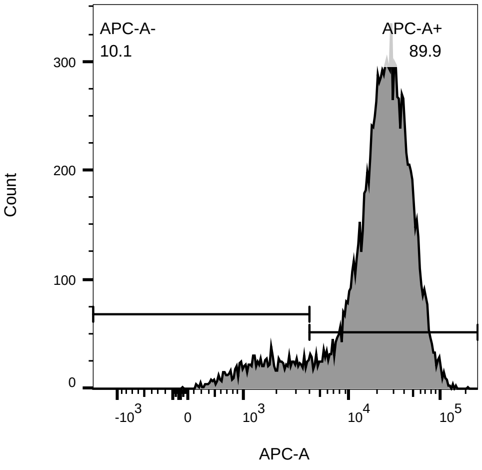

LCL472_CD46KO-1_015.fcs
Single Cells
14187

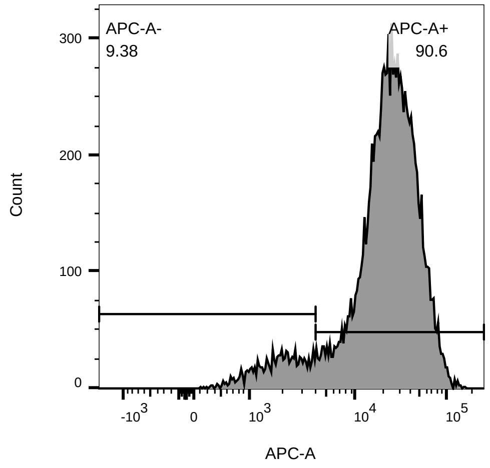

LCL472_CD46+SCDKO-2_018.fcs
Single Cells
13524

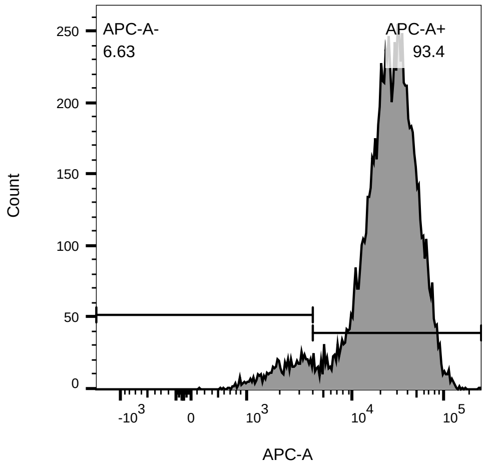

LCL472_CD46+SCDKO-1_017.fcs
Single Cells
10757

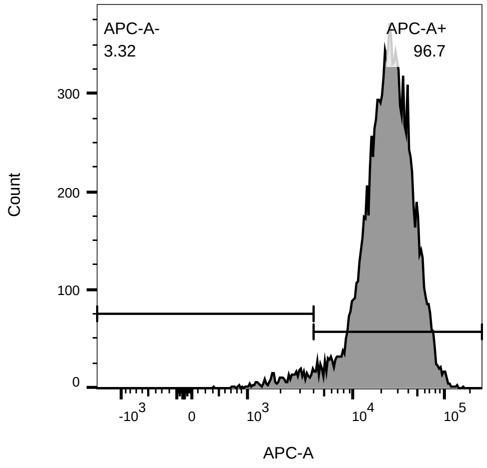

LCL472_CD46+MDM2KO-2_024.fcs
Single Cells
14134

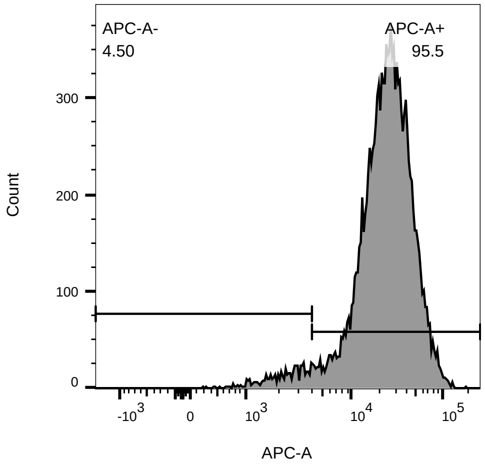

LCL472_CD46+MDM2KO-1_023.fcs
Single Cells
14428

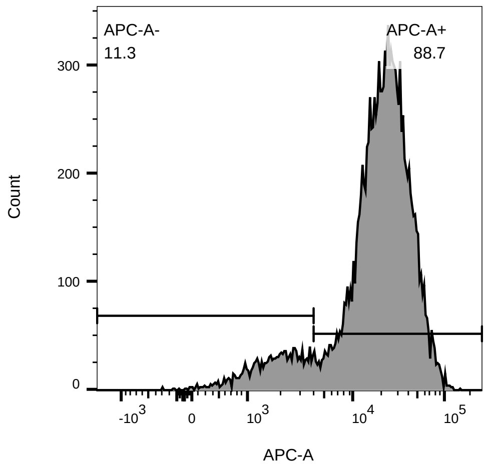

LCL472_CD46+FADS2KO-2_020.fcs
Single Cells
14680

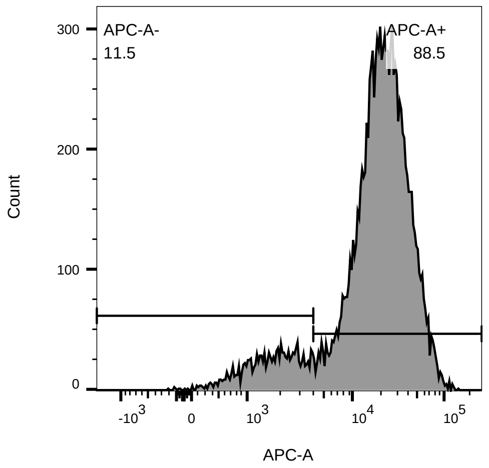

LCL472_CD46+FADS2KO-1_019.fcs
Single Cells
13934

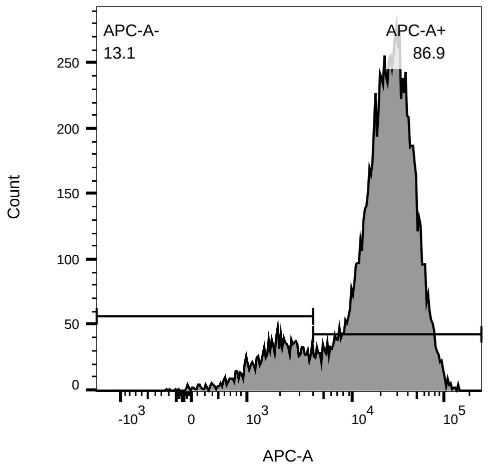

LCL472_CD46+FADS2+SCDKO-2_022.fcs
Single Cells
12919

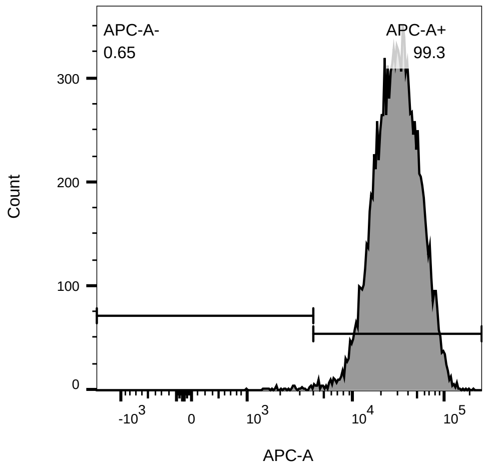

LCL472_CD46+FADS2+SCDKO-1_021.fcs
Single Cells
13373

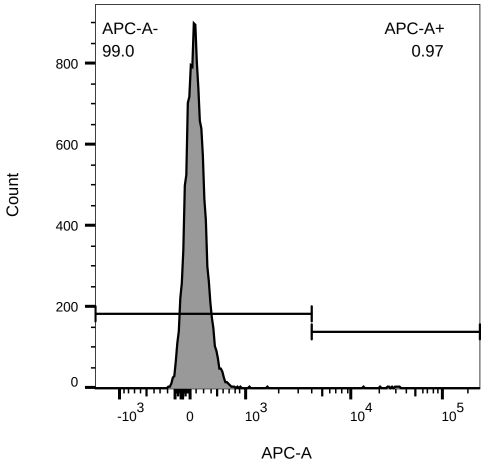

LCL1303_Unstained_Untransfected_001.fcs
Single Cells
13849

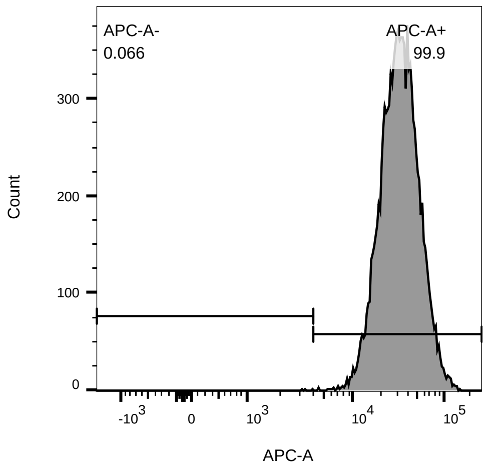

LCL1303_Stained_Untransfected_002.fcs
Single Cells
12116

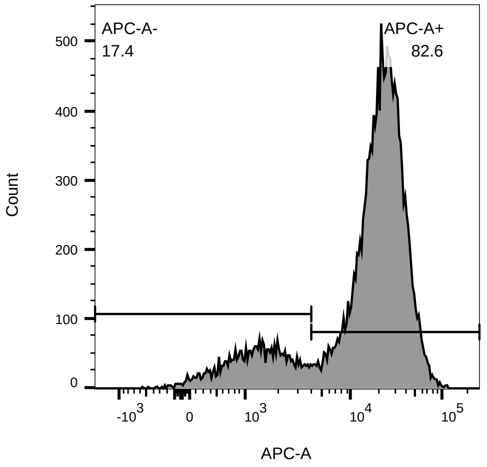

LCL1303_CD46KO-2_004.fcs
Single Cells
20700

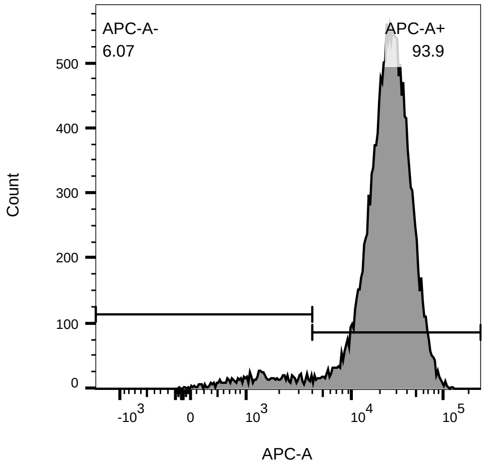

LCL1303_CD46KO-1_003.fcs
Single Cells
20204

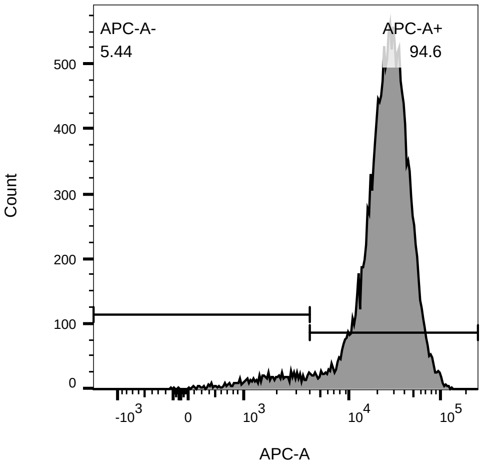

LCL1303_CD46+SCDKO-2_006.fcs
Single Cells
19886

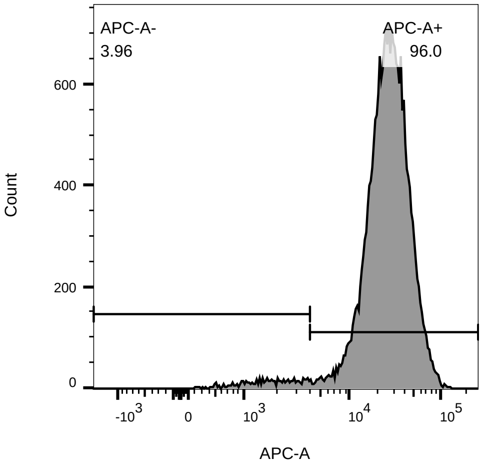

LCL1303_CD46+SCDKO-1_005.fcs
Single Cells
24574

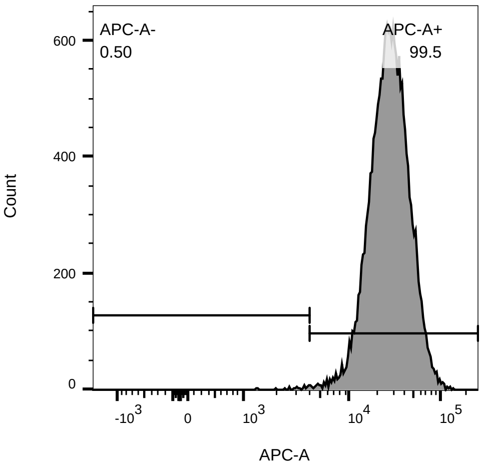

LCL1303_CD46+MDM2KO-2_012.fcs
Single Cells
20566

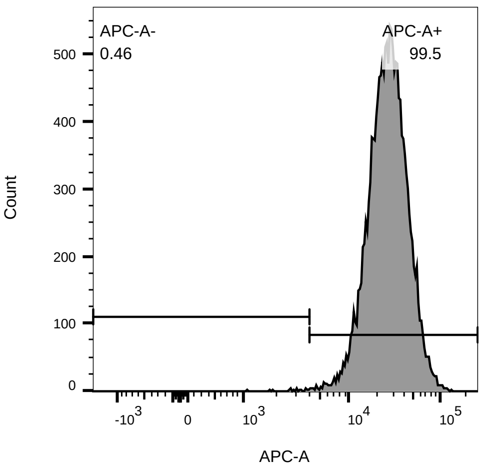

LCL1303_CD46+MDM2KO-1_011.fcs
Single Cells
17045

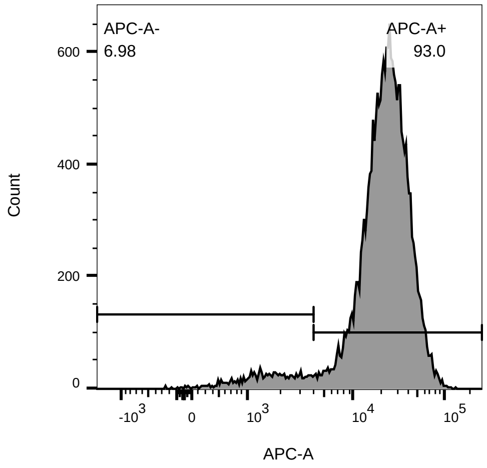

LCL1303_CD46+FADS2KO-2_008.fcs
Single Cells
23091

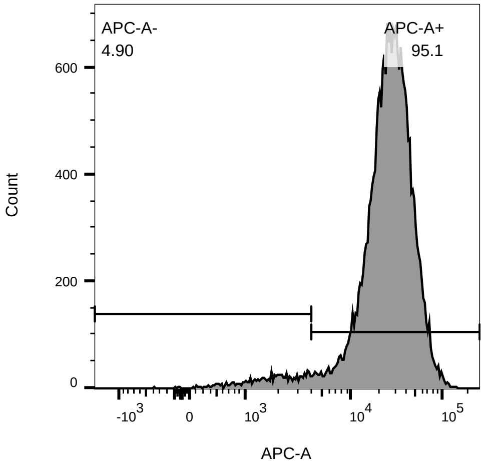

LCL1303_CD46+FADS2KO-1_007.fcs
Single Cells
24700

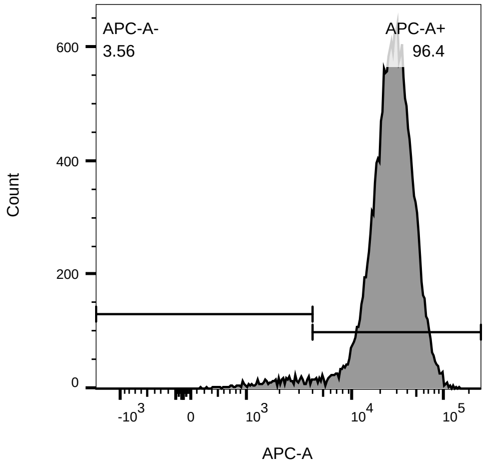

LCL1303_CD46+FADS2+SCDKO-2_010.fcs
Single Cells
21453

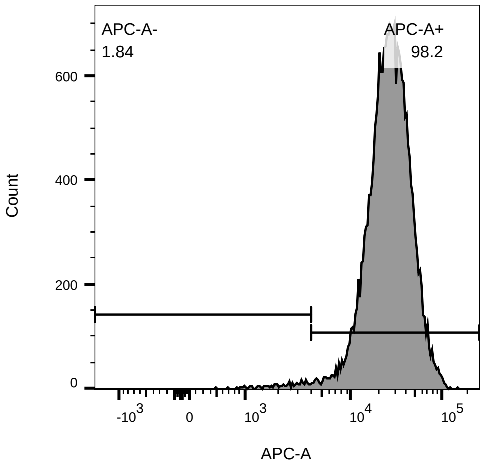

LCL1303_CD46+FADS2+SCDKO-1_009.fcs
Single Cells
24439
