# Supplementary material for: Fatty acid desaturases link cell metabolism pathways to promote proliferation of Epstein-Barr virus-infected B cells
Source: PLoS Pathog. 2025 May 22;21(5):e1012685. doi: 10.1371/journal.ppat.1012685 (PMC12143519; doi:10.1371/journal.ppat.1012685)
Supplement: S3 File — (A) Gating strategy corresponding to flow cytometry data displayed in Fig 3B, C. (B) Complete flow cytometry dot plots at 3 days post transfection, corresponding to Fig 3B, C. (C) Complete flow cytometry dot plots at 10 days post transfection, corresponding to Fig 3B, C. (D) Raw data corresponding to Fig 3D. (E) Uncropped Western blot corresponding to Fig 3E. (ZIP) [file ppat.1012685.s009.zip › S3_File/C_Fig3_B-day10.pdf]

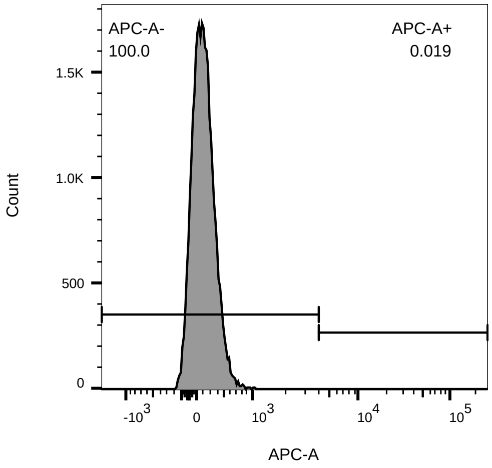

LCL472_Unstained_Untransfected_013.fcs
Single Cells
32232

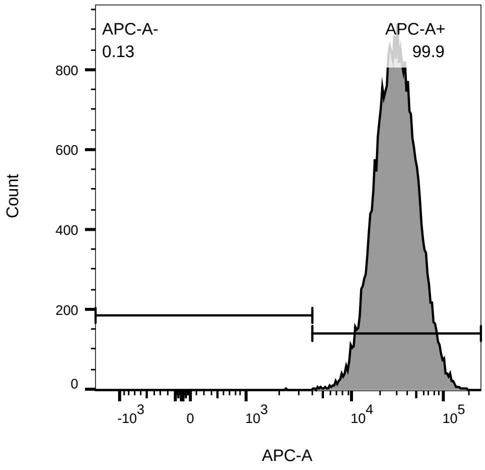

LCL472_Stained_Untransfected_014.fcs
Single Cells
32521

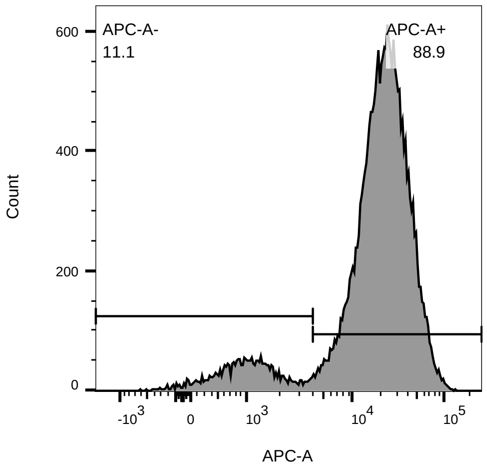

LCL472_CD46KO-1_015.fcs
Single Cells
26403

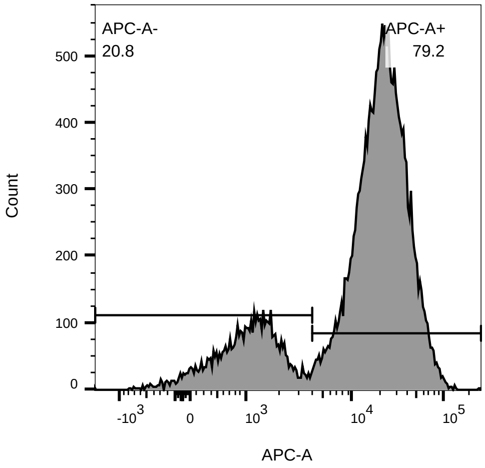

LCL472_CD46KO-2_016.fcs
Single Cells
27360

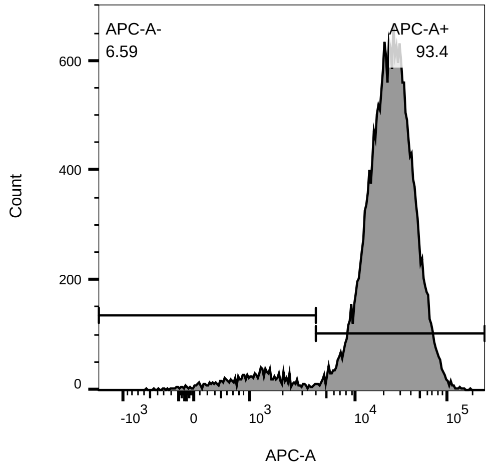

LCL472_CD46+SCDKO-2_018.fcs
Single Cells
26279

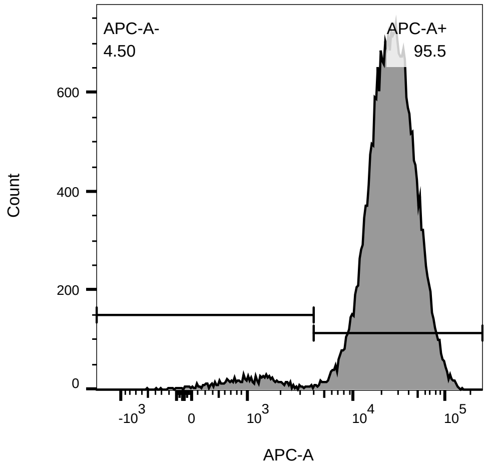

LCL472_CD46+SCDKO-1_017.fcs
Single Cells
30900

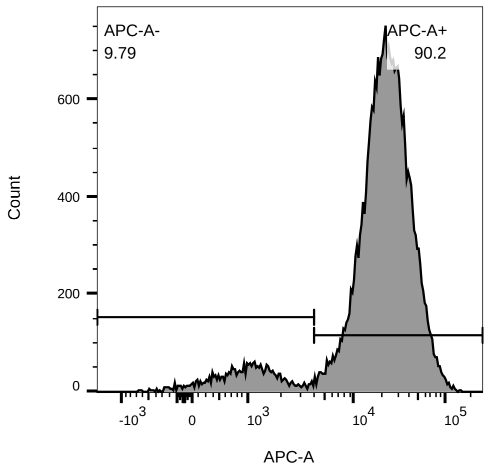

LCL472_CD46+FADS2KO-1_019.fcs
Single Cells
31620

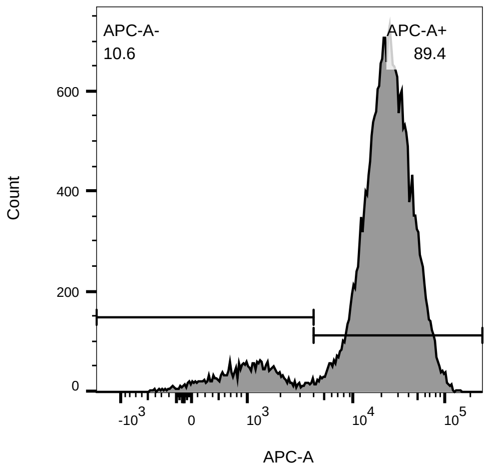

LCL472_CD46+FADS2KO-2_020.fcs
Single Cells
30683

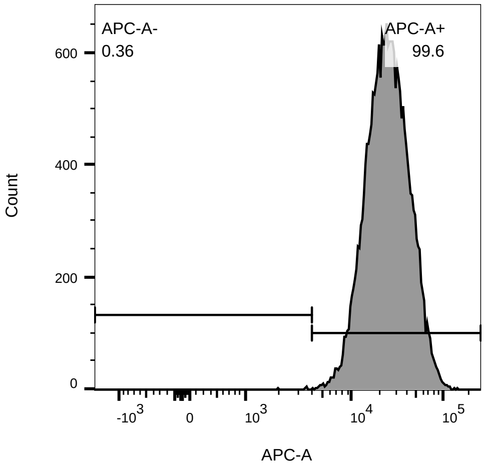

LCL472_CD46+FADS2+SCDKO-1_021.fcs
Single Cells
23842

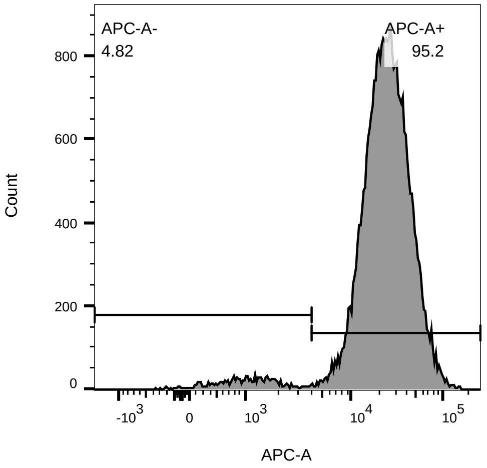

LCL472_CD46+FADS2+SCDKO-2_022.fcs
Single Cells
34416

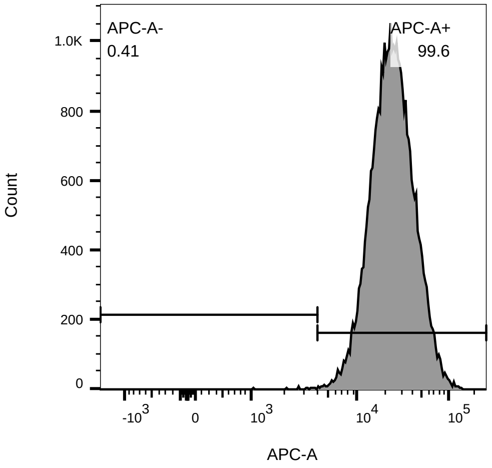

LCL472_CD46+MDM2KO-1_023.fcs
Single Cells
36967

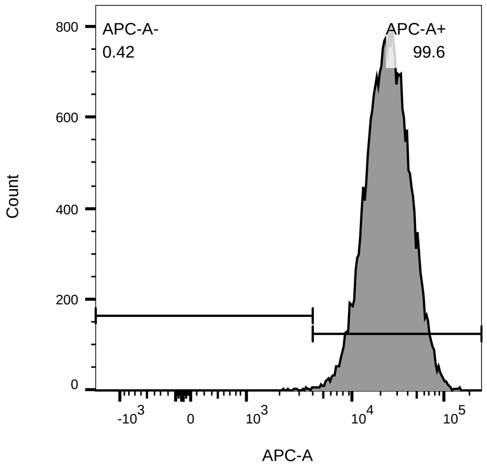

LCL472_CD46+MDM2KO-2_024.fcs
Single Cells
29900

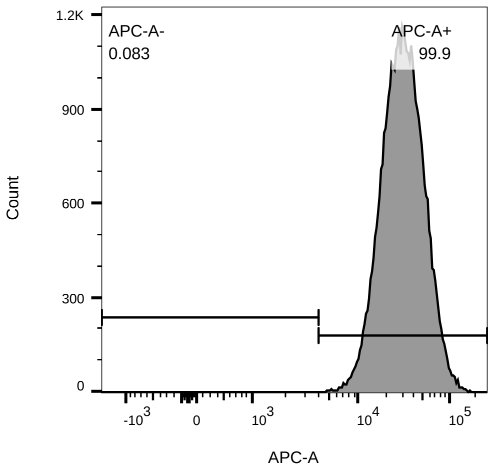

LCL1303_Stained_Untransfected_002.fcs
Single Cells
42156

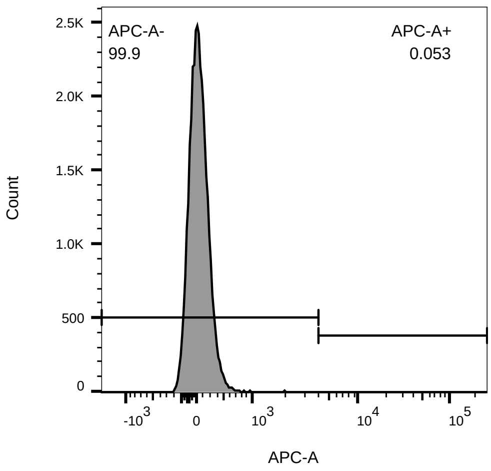

LCL1303_Unstained_Untransfected_001.fcs
Single Cells
37893

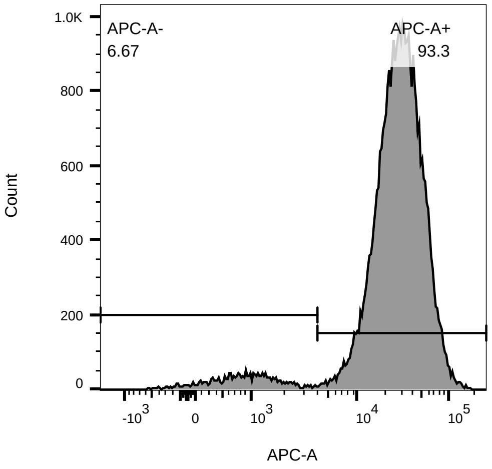

LCL1303_CD46KO-1_003.fcs
Single Cells
39972

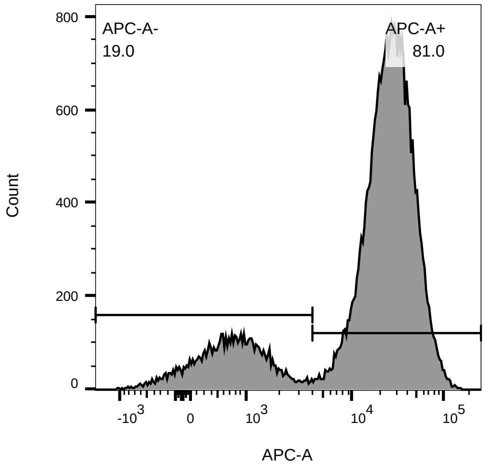

LCL1303_CD46KO-2_004.fcs
Single Cells
38017

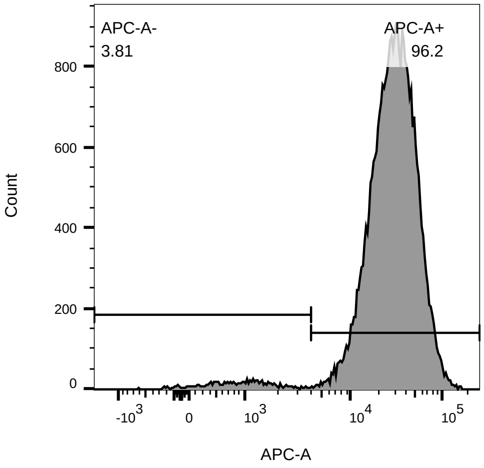

LCL1303_CD46+SCDKO-1_005.fcs
Single Cells
35786

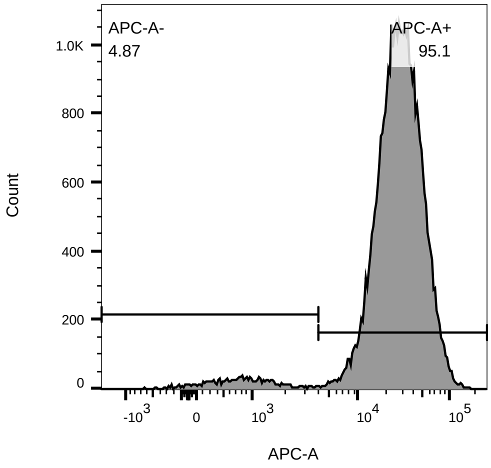

LCL1303_CD46+SCDKO-2_006.fcs
Single Cells
42149

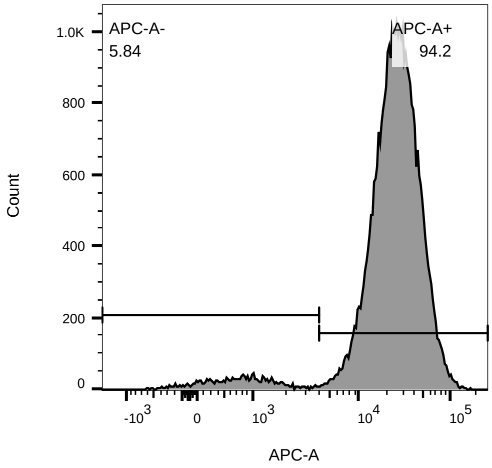

LCL1303_CD46+FADS2KO-1_007.fcs
Single Cells
40663

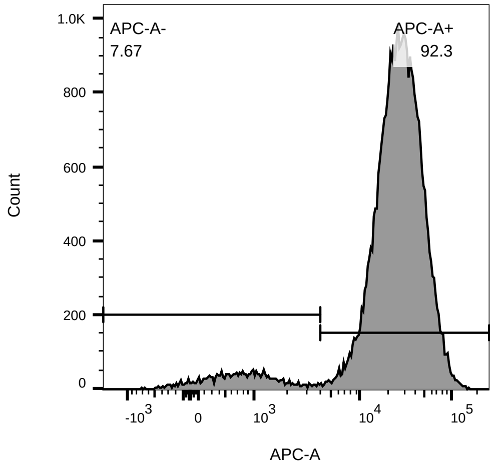

LCL1303_CD46+FADS2KO-2_008.fcs
Single Cells
40105

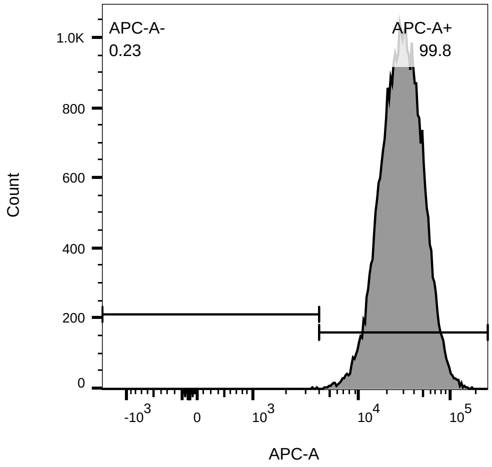

LCL1303_CD46+MDM2KO-1_011.fcs
Single Cells
37820

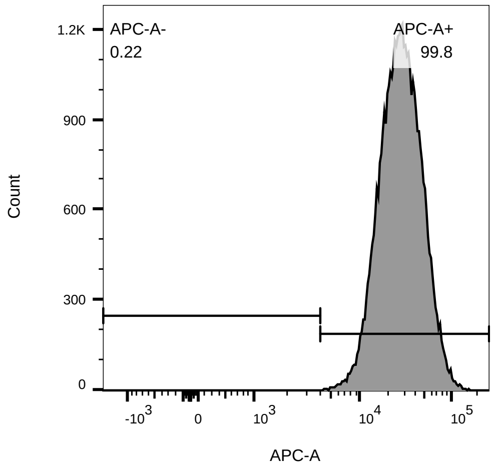

LCL1303_CD46+MDM2KO-2_012.fcs
Single Cells
43743

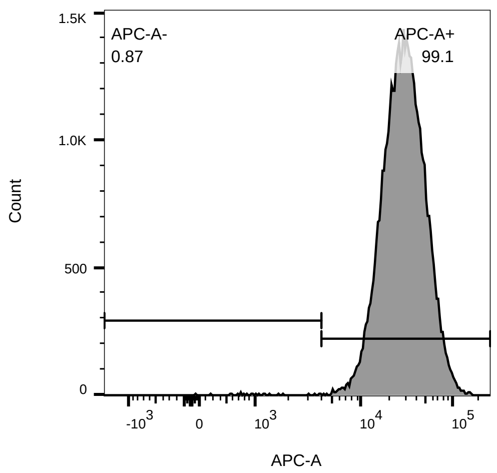

LCL1303_CD46+FADS2+SCDKO-1_009.fcs
Single Cells
51108

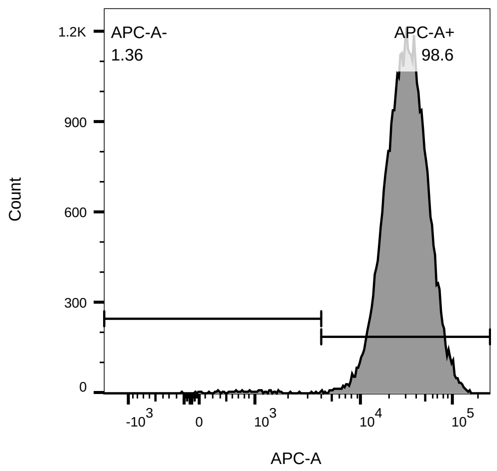

LCL1303_CD46+FADS2+SCDKO-2_010.fcs
Single Cells
43095
