# Supplementary material for: Fatty acid desaturases link cell metabolism pathways to promote proliferation of Epstein-Barr virus-infected B cells
Source: PLoS Pathog. 2025 May 22;21(5):e1012685. doi: 10.1371/journal.ppat.1012685 (PMC12143519; doi:10.1371/journal.ppat.1012685)
Supplement: S4 File — (A) Raw data corresponding to Fig 4B, C. (B) Gating strategy corresponding to flow cytometry data in Fig 4D, E. (C) Complete flow cytometry dot plots corresponding to Fig 4D, E. (D) Raw data corresponding to Fig 4D, E. (E) Raw data corresponding to Fig 4F. (F) Raw data corresponding to Fig 4G. (G) Gating strategy for Annexin-V measurement, corresponding to Fig 4H. (H) Gating strategy for cleaved caspase 3/7 measurement, corresponding to Fig 4H. (I) Raw data corresponding to Fig 4H. (ZIP) [file ppat.1012685.s010.zip › S4_File/G_Fig4H_Annexin.pdf]

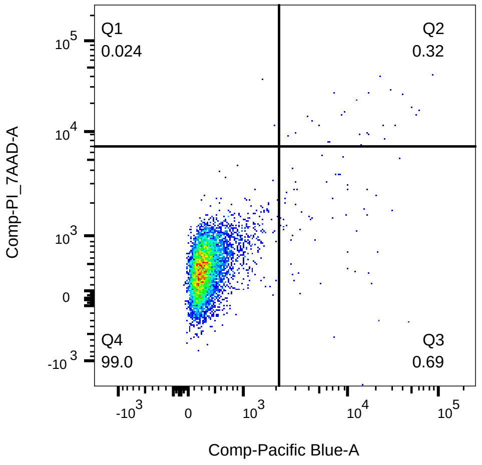

1303_DMSO-BSA-1.fcs
Single Cells
8224

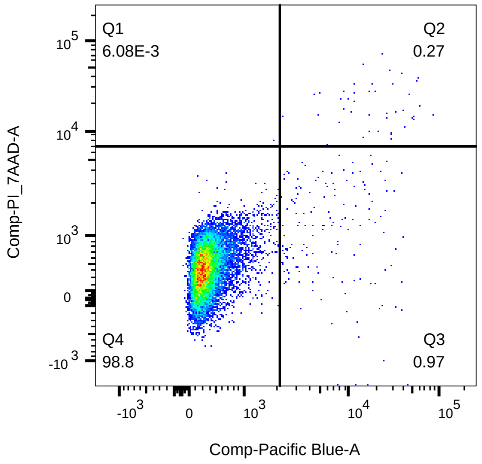

1303_DMSO-BSA-2.fcs
Single Cells
16455

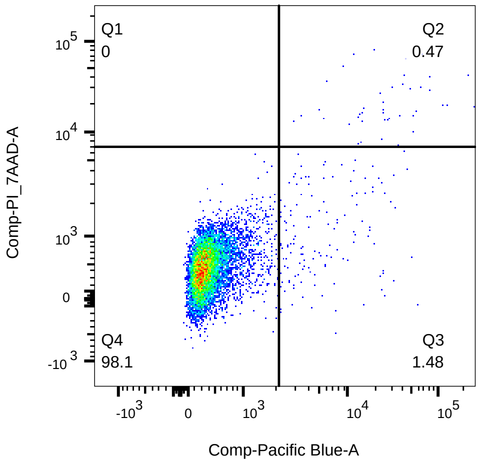

1303_DMSO+100Pal-1.fcs
Single Cells
9152

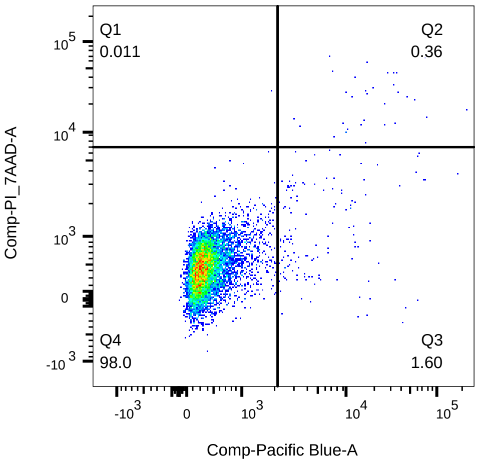

1303_DMSO+100Pal-2.fcs
Single Cells
8789

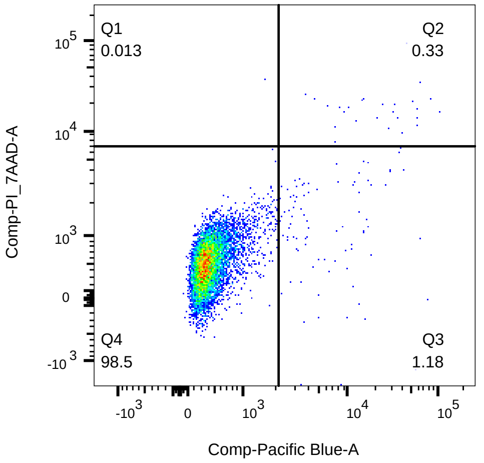

1303_FADS2i-BSA-1.fcs
Single Cells
7984

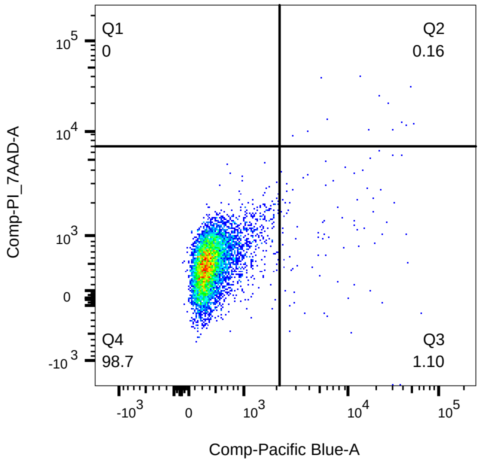

1303_FADS2i-BSA-2.fcs
Single Cells
8115

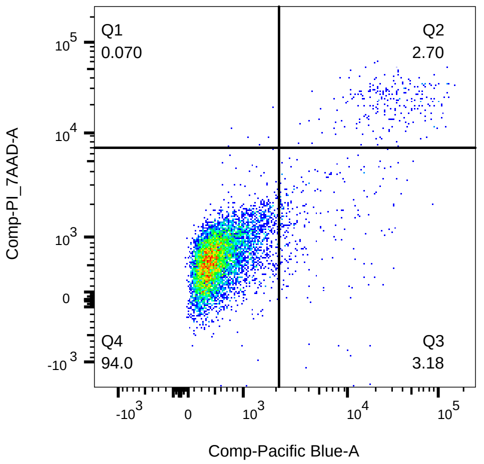

1303_FADS2i+100Pal-1.fcs
Single Cells
8547

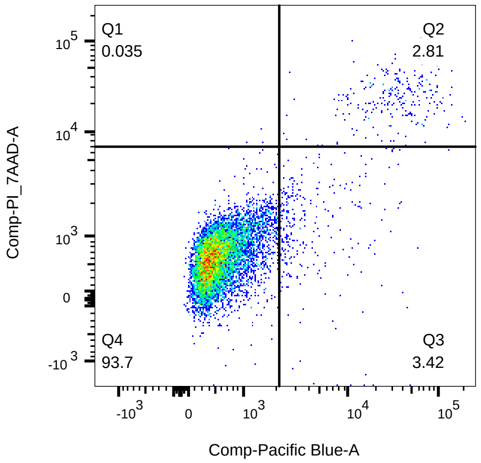

1303_FADS2i+100Pal-2.fcs
Single Cells
8547

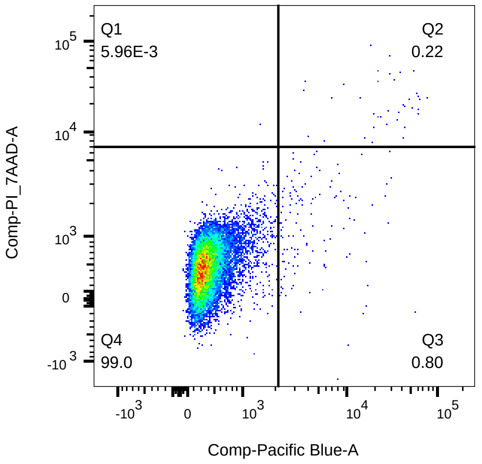

1303_SCDi-BSA-1.fcs
Single Cells
16769

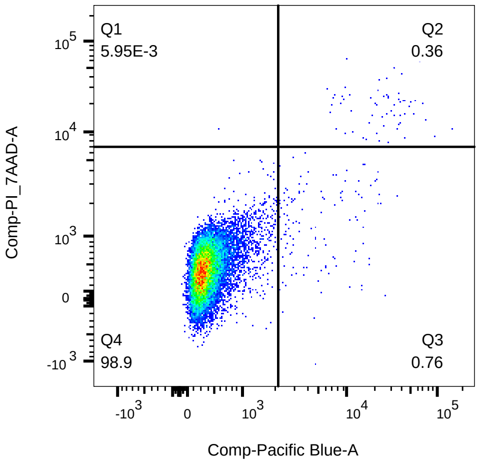

1303_SCDi-BSA-2.fcs
Single Cells
16817

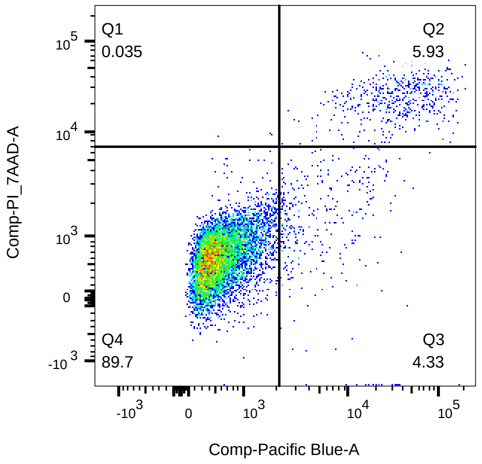

1303_SCDi+100Pal-2.fcs
Single Cells
8479

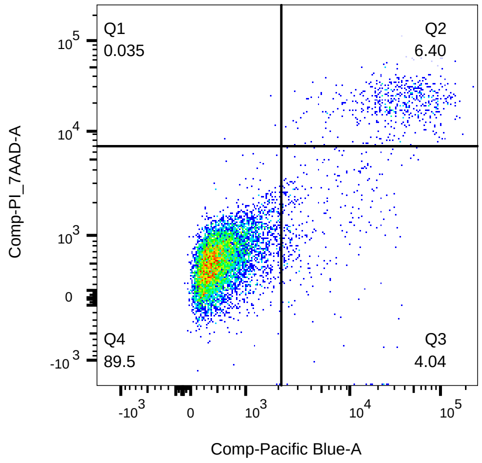

1303_SCDi+100Pal-1.fcs
Single Cells
8481

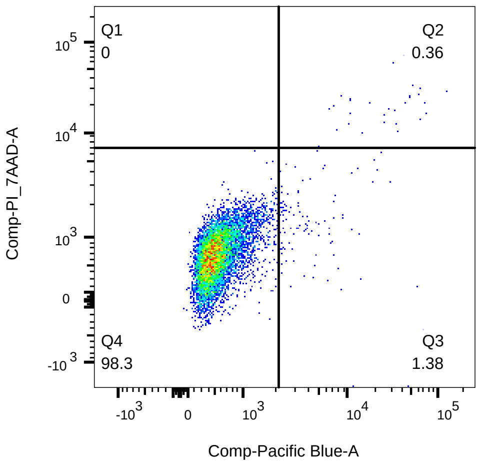

1303_SCDi+FADS2i+BSA-1.fcs
Single Cells
8108

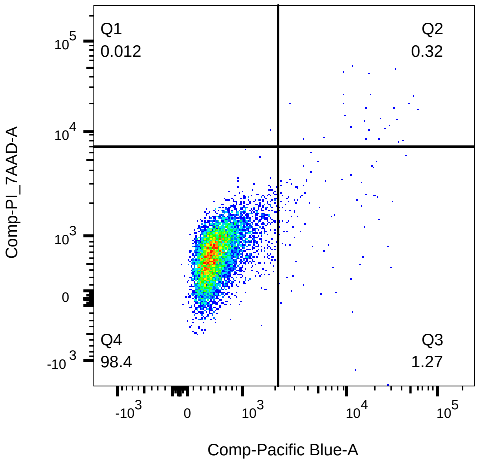

1303_SCDi+FADS2i+BSA-2.fcs
Single Cells
8342

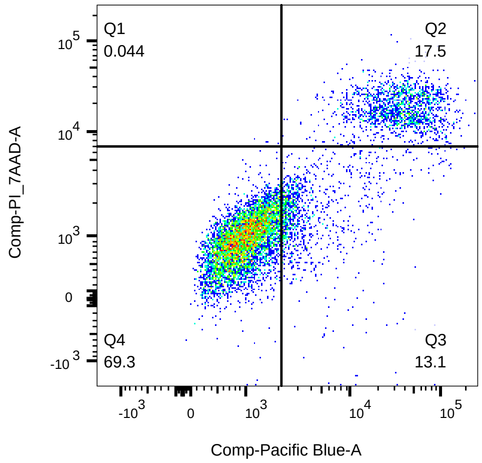

1303_SCDi+FADS2i+100Pal-1.fcs
Single Cells
9088

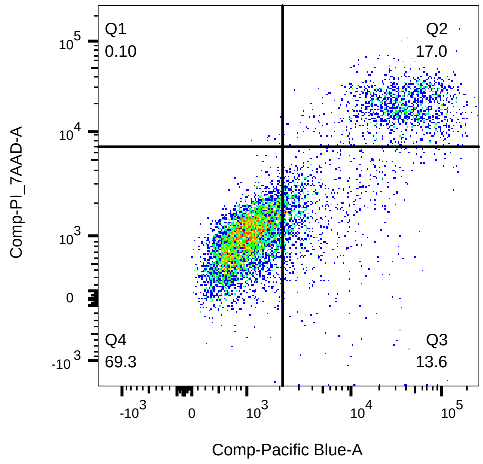

1303_SCDi+FADS2i+100Pal-2.fcs
Single Cells
7984

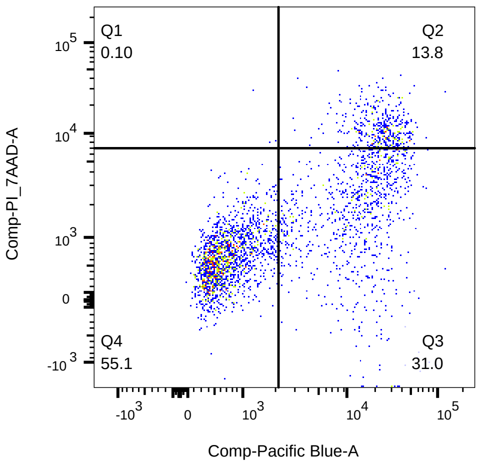

1303_200-Pan-1.fcs
Single Cells
2885

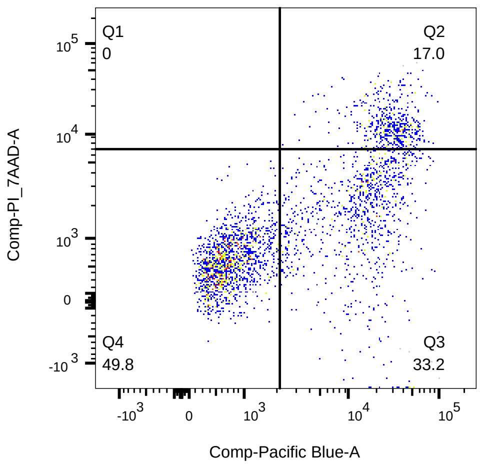

1303_200-Pan-2.fcs
Single Cells
2788

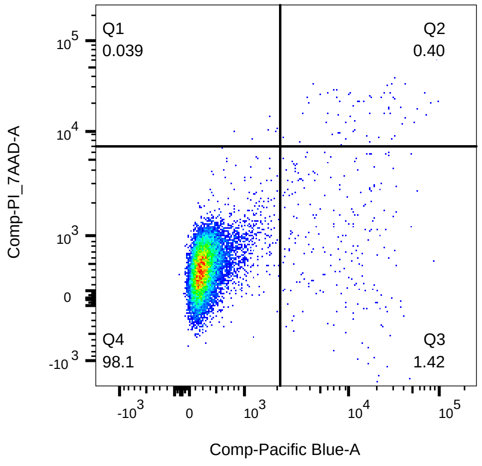

1343_DMSO-BSA-1.fcs
Single Cells
15522

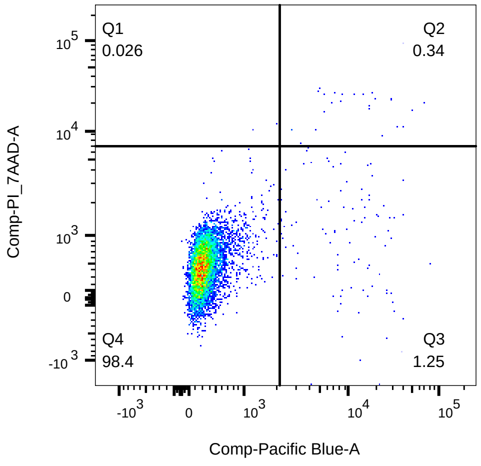

1343_DMSO-BSA-2.fcs
Single Cells
7736

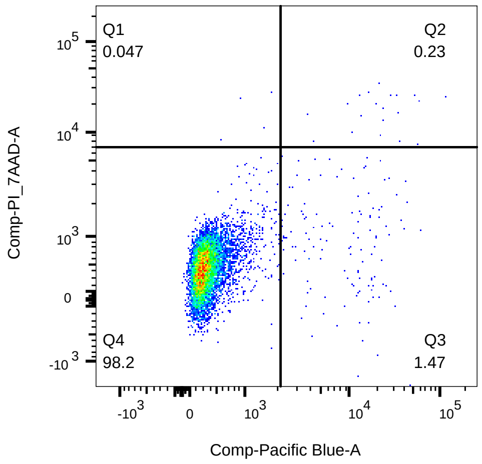

1343_DMSO+100Pal-1.fcs
Single Cells
8562

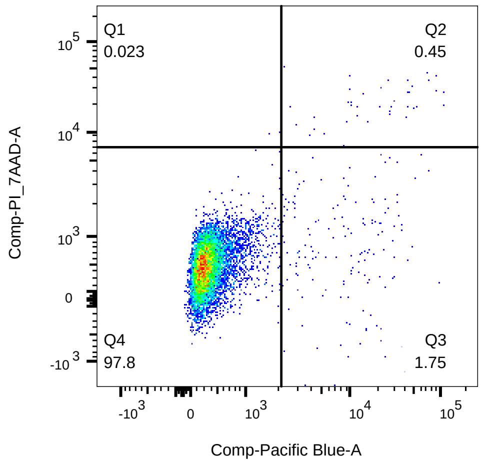

1343_DMSO+100Pal-2.fcs
Single Cells
8734

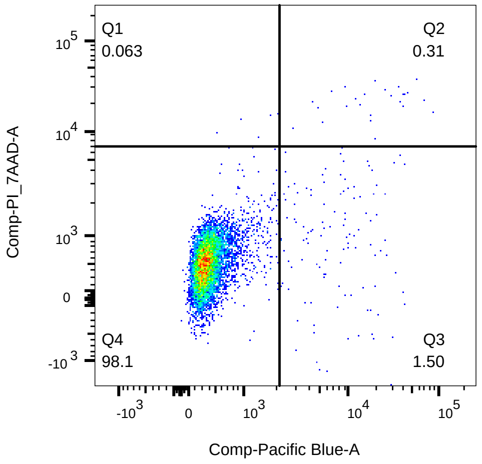

1343_FADS2i-BSA-1.fcs
Single Cells
7997

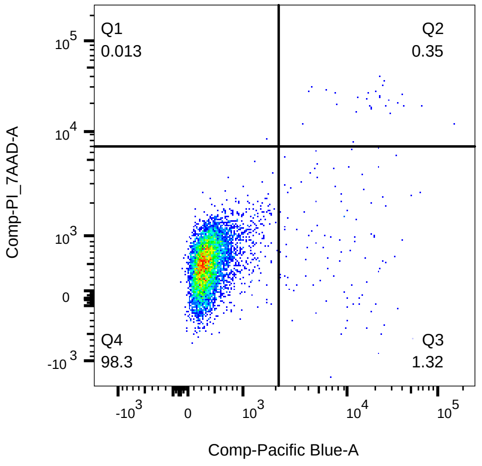

1343_FADS2i-BSA-2.fcs
Single Cells
7955

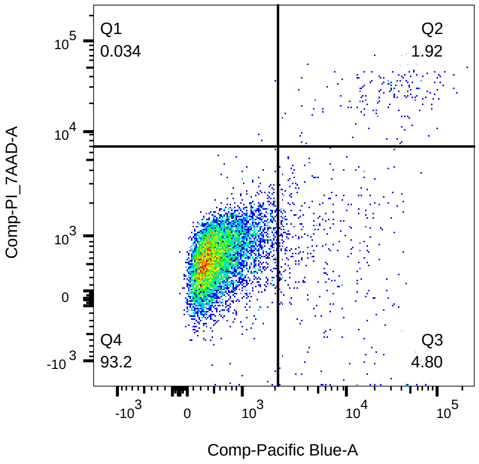

1343_FADS2i+100Pal-1.fcs
Single Cells
8798

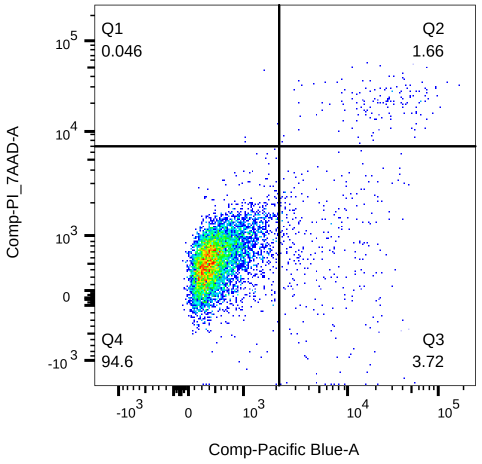

1343_FADS2i+100Pal-2.fcs
Single Cells
8663

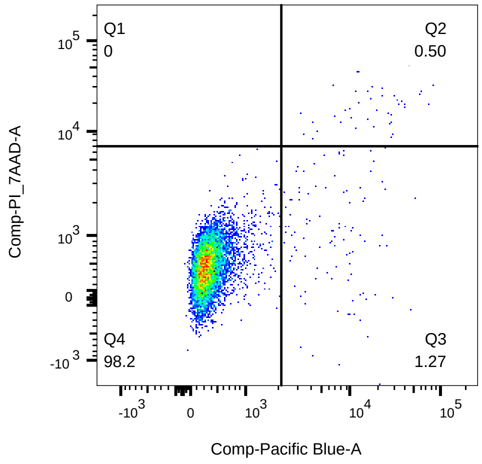

1343_SCDi-BSA-1.fcs
Single Cells
8264

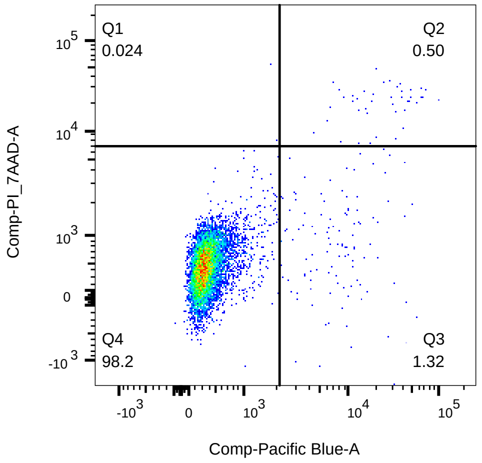

1343_SCDi-BSA-2.fcs
Single Cells
8334

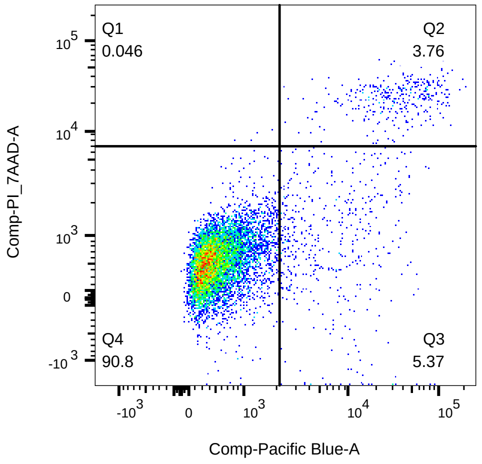

1343_SCDi+100Pal-1.fcs
Single Cells
8782

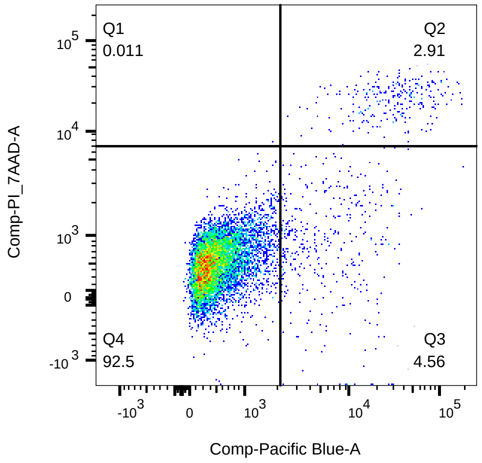

1343_SCDi+100Pal-2.fcs
Single Cells
8802

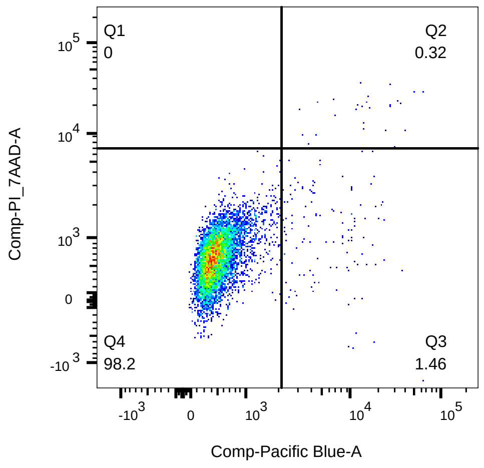

1343_SCDi+FADS2i+BSA-1.fcs
Single Cells
8192

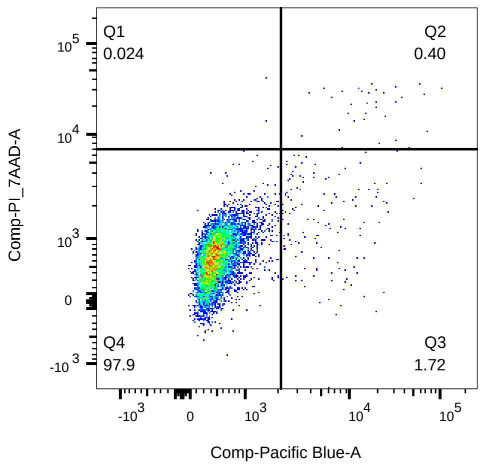

1343_SCDi+FADS2i+BSA-2.fcs
Single Cells
8315

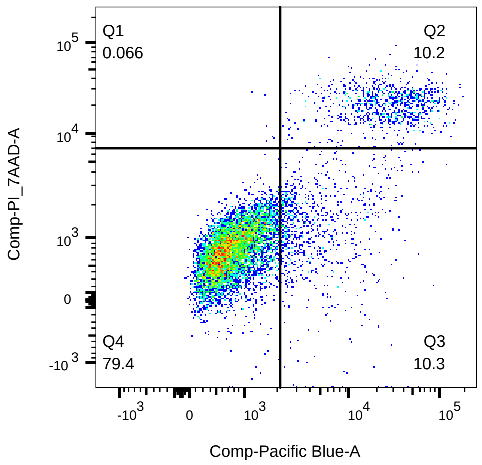

1343_SCDi+FADS2i+100Pal-1.fcs
Single Cells
9072

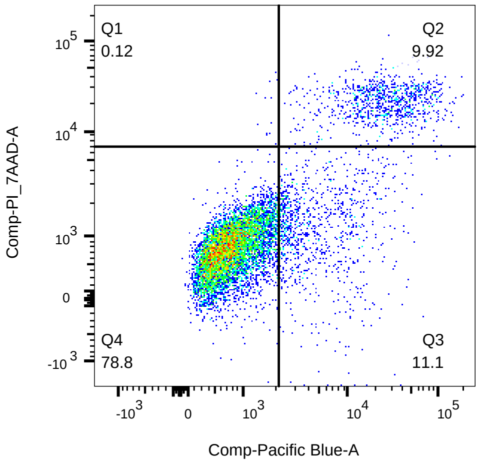

1343_SCDi+FADS2i+100Pal-2.fcs
Single Cells
9069

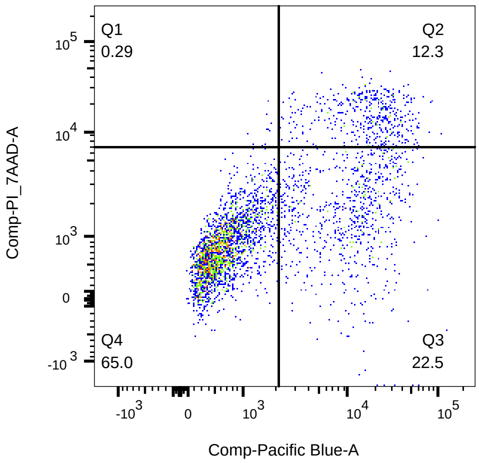

1343_200-Pan-1.fcs
Single Cells
3416

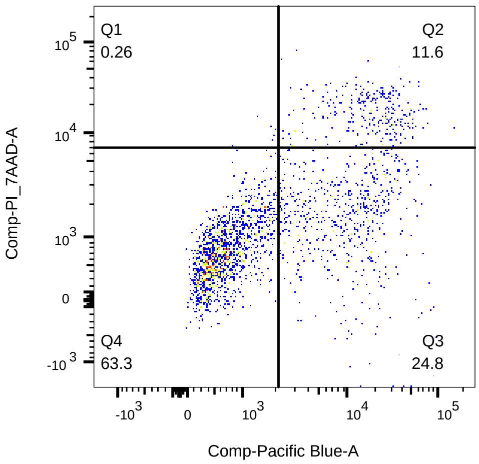

1343_200-Pan-2.fcs
Single Cells
2289
