# Supplementary material for: Fatty acid desaturases link cell metabolism pathways to promote proliferation of Epstein-Barr virus-infected B cells
Source: PLoS Pathog. 2025 May 22;21(5):e1012685. doi: 10.1371/journal.ppat.1012685 (PMC12143519; doi:10.1371/journal.ppat.1012685)

Uncropped Western blot of EBV-infected B cells throughout early infection, corresponding to S1 Fig.

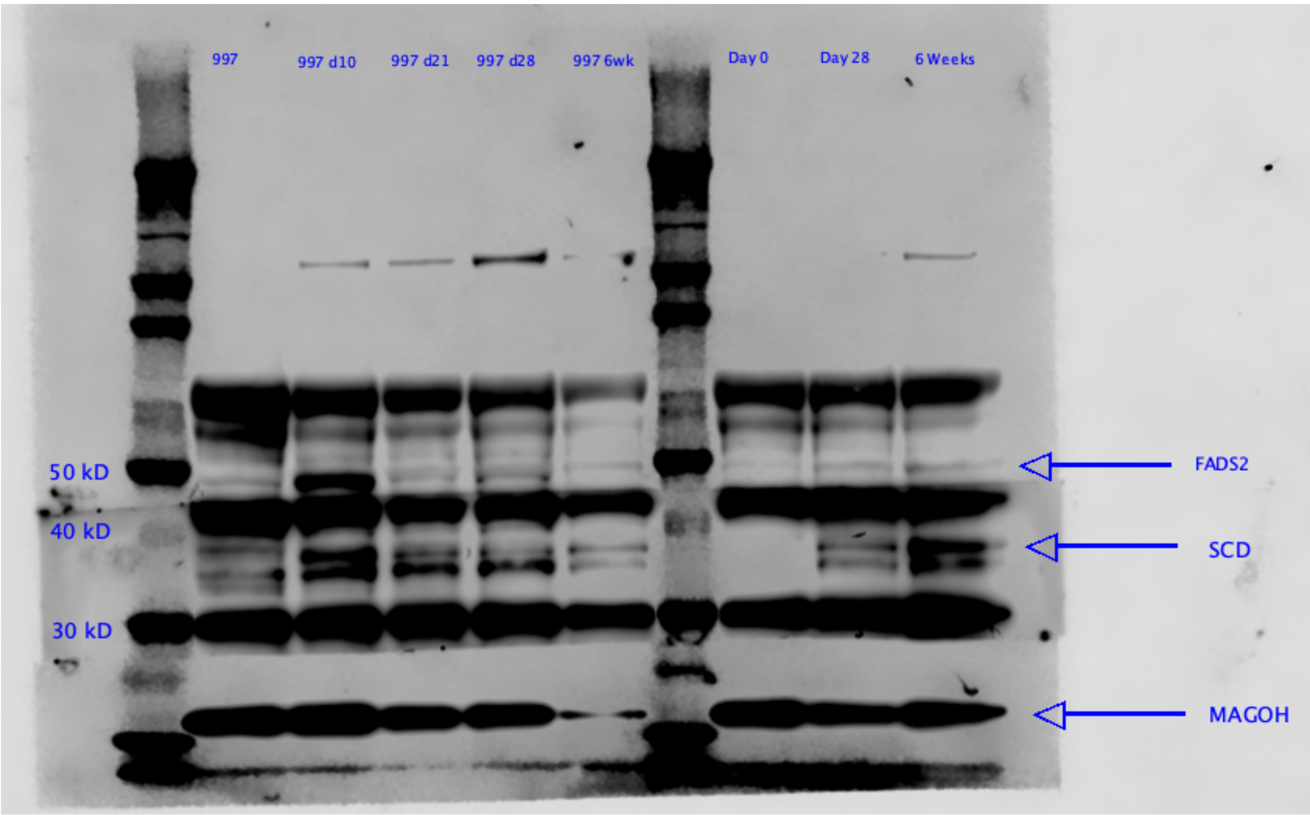

Supplement: S7 File — (A) Uncropped Western blot corresponding to S1 Fig. (B) Raw data corresponding to S2 Fig. (C) Raw data corresponding to S3 Fig. (ZIP) [file ppat.1012685.s013.zip › S7_File/A_S1Fig.pdf]
