# Supplementary material for: Fatty acid desaturases link cell metabolism pathways to promote proliferation of Epstein-Barr virus-infected B cells
Source: PLoS Pathog. 2025 May 22;21(5):e1012685. doi: 10.1371/journal.ppat.1012685 (PMC12143519; doi:10.1371/journal.ppat.1012685)
Supplement: Attachment — Submitted filename: Response-to-reviewer-comments-SCD_FADS2_030225_ml.docx [file ppat.1012685.s015.docx]

**“Fatty acid desaturases link cell metabolism pathways to promote proliferation of Epstein-Barr virus-infected B cells”: Rebuttal letter**

We would like to thank the editor and reviewers for the opportunity to revise our manuscript. We also greatly appreciate the constructive feedback provided by the reviewers. After incorporating the requested revisions, we feel that the quality of work and strength of our argument has improved.

**Editorial Comments:**

1. *Line 89: are there only three human fatty acid desaturases? Please confirm.*

**Response:** Thank you for the comment. We have clarified this in the manuscript and added an additional reference. In humans, there are three desaturases acting on fatty acids: Δ6 desaturase (FADS2), Δ5 desaturase (FADS1) and Δ9 desaturase (SCD) [1]. There is an additional Δ4 desaturase that acts primarily on membrane sphingolipids and is involved in ceramide metabolism [2]. However, this minor desaturase is not implicated in the central fatty acid metabolism pathways assessed in our study.

2. *Lines 93-96: please provide the reference for this sentence.*

**Response:** Thank you for pointing this out. We have added the reference.

3. *Line 432: Please define UFA as unsaturated fatty acid.*

**Response:** Thank you for the comment. We have clarified this in the manuscript.

4. *Line 539: Did they mean palmitoleate instead of oleate?*

**Response:** Thank you for notifying us. We meant palmitoleate, and we have corrected this.

5. *Reference 43 is a duplicate of reference 38. Same with reference 46/55. The other references should be checked.*

**Response:** Thank you for notifying us that we had duplicate references. These duplicates were due to improper merging of references when libraries were shared between coauthors. We have removed those and checked the entire reference list for additional duplicates.

**Reviewer #1:**

1. *The key observations in the presented study should be tested in the context of primary uninfected B cells stimulated to proliferate physiologically (BCR crosslinking, CD40 or TLR ligation, etc) to determine the extent to which the observed phenotypes are modified by EBV.*

**Response:** We thank the reviewer for this suggestion, and we agree that this would be valuable addition to our study. To our knowledge, the combined role of SCD1 and FADS2 had not yet been studied in uninfected, primary B cells. To address this, we stimulated PBMCs with either CpG or recombinant CD40L+IL-4 in the presence of cyclosporine A. Simultaneously, we treated stimulated PBMCs with SCD1i and/or FADS2i. We found that inhibition of either SCD1 or FADS2 decreased the number of CD19+ B cells that proliferated by day five in both stimulation conditions (**Figure 1A-C below and new Figure 2J-K and Supplementary Figure 4**). However, similar to LCLs, treatment with both SCD1i and FADS2i simultaneously further decreased the number of CD19+ B cells that proliferated in response to these mitogens. These data suggest that the EBV-driven SCD1/FADS2 upregulation and fatty acid desaturation help fulfill the metabolic requirements needed for B cells to divide.

**Figure 1: Stimulated uninfected B cells also require SCD1 and FADS2 for proliferation. A**) Representative flow cytometry histograms five days post stimulation, showing dilution of CellTrace Violet proliferation dye. Counts are normalized to mode. **B**) Numbers of CD19+, CellTrace Violet-low cells five days post CpG stimulation. Cell numbers were normalized to counting beads and displayed as a percentage of DMSO (n=4). **C**) Numbers of CD19+, CellTrace Violet-low cells five days post recombinant CD40L+IL-4 stimulation. Cell numbers were normalized to counting beads and displayed as a percentage of DMSO (n=4).

2. *It is not clear whether the key phenotypes were tested in LCLs derived from more than one independent donors. The inter-donor variability should be shown.*

**Response:** We thank the reviewer for pointing out that the number of donors used in each experiment is not clear. We have added this information to each figure legend. Each experiment used LCLs from 2-4 different donors.

3. *It is not clear whether the experimental manipulation of SCD1 and FADS2 activity and expression has an effect on EBV gene expression and latent/lytic cycle*

**Response:** We tested whether SCD1i+FADS2i results in changes in latent EBV gene expression and/or lytic gene expression using real-time qPCR **(Figure 2 below)**, and the results section has been updated to reflect these findings (added as **Supplementary Figure 1**). In brief, we found lower expression of EBNA1 and EBNA3A after desaturase inhibition, which we think may be effects of the altered cell cycle dynamics due to desaturase inhibition (especially since EBNA1 mRNA abundance is highest during S phase, and desaturase inhibition decreases the percentage of cells in S phase [3]). Additionally, these changes in EBNA1 and EBNA3A expression occurred after 72hrs SCD1i+FADS2i treatment, while the growth arrest phenotype was strongest after 48hrs treatment with the inhibitors. Therefore, it is unlikely that changes in expression of EBNA1 and EBNA3A are driving the growth arrest phenotype. While we were able to detect an upregulation of lytic genes (BZLF1 and BMRF1) in our positive control samples (P3HR1-ZHT cells treated with either vehicle or 4-hydroxytamoxifen), we did not find a significant upregulation of these genes in LCLs after desaturase inhibition. Together, these data suggest that SCD1i+FADS2i primarily modulate host B cell, and not viral, factors.

**Figure 2: Desaturase-dependent growth arrest does not involve changes in EBV gene expression.** **A-E**) qPCR data showing relative expression of latency-associated EBV genes, relative to DMSO. **F-I**) qPCR data showing relative expression of lytic-associated EBV genes, relative to DMSO. The ZHT cell line treated with 4-hydroxytamoxifen (4HT) to induce lytic reactivation was included as a positive control.

4. *SCD1 and FADS2 regulation of fatty acid saturation has been linked to ferroptosis, ER stress response, and eicosanoid signaling, in addition to the expected link to metabolism. While ER stress is touched upon in the discussion, the discussion needs to be fleshed out to include additional potential mechanisms and their fit with the observed phenotypes.*

**Response:** We thank the reviewer for this suggestion. We have expanded on our discussion of the potential mechanism driving the SCD1i/FADS2i-mediated cell cycle arrest to include evidence of lipolysis and docosahexaenoic acid signaling in T cells after SCD1 inhibition and the role of free fatty acid profile remodeling in preventing ferroptosis. Because LCLs are not dying when SCD1 and FADS2 are inhibited, ferroptosis can be ruled out as a mechanism behind our observed phenotypes. Additionally, FADS2 inhibition often suppresses rather than induces ferroptosis [4,5]. However, it remains possible that the increase in free PUFAs after SCD1/FADS2 inhibition helps prevent ferroptosis, especially given the observed mitochondrial dysfunction, because ferroptosis is typically driven by peroxidation of membrane (i.e. esterified) and not free PUFAs [6].

5. *Apoptosis assays in Fig. 2H do not have a positive control*

**Response:** We have replaced this panel with a simpler depiction of the lack of cell death induced by SCD1i+FADS2i treatment using absolute cell counts and percentage Trypan - (i.e. alive) normalized to DMSO. With this panel (**Figure 3 below and new Figure 2H**), it is clear that the effect the inhibitors have on live cell number cannot be explained by increased cell death. Therefore, the reduction in live cell number is instead due to cell cycle arrest. Our results have been updated accordingly.

**Figure 3: SCD1 and FADS2 inhibition does not induce cell death.** Live Cell % indicates the percentage of viable (i.e. Trypan negative) in the population. Live cell number indicates the absolute number of live cells (Trypan negative). Both live cell % and absolute number presented as normalized values relative to DMSO.

6. *Fig. 3 traces proportion of targeted LCLs in a mixed cell culture over 10 days. The conclusion that is made refers to decreased cell proliferation in targeted cells, but there are no data to support this claim, as multiple factors can affect the change in proportion of targeted vs. not cells during culture*

**Response:** We have also provided numbers of targeted cells cultured over a ten-day period (**Figure 4 below and new Figure 3D in the manuscript**). The cell numbers are normalized to counting beads and multiplied by the dilution factor each time the cells were split. The cell numbers are consistent with our data showing the percentage of cells transfected, now in **Figure 3C** of the manuscript, and consistent with our interpretation that simultaneous SCD1+FADS2 targeting decreases proliferation. We agree that multiple factors can affect the change in proportion of targeted vs. not targeted cells in culture. As such, we included a Cas9-RNP complex targeting MDM2 as a positive control, since removing this negative regulator of p53 is lethal in LCLs [7].

**Figure 4: Simultaneous genetic targeting of SCD1 and FADS2 decreases LCL proliferation.** CD46- cells were counted at each timepoint and normalized to counting beads. All data shown relative to initial normalized counts three days post transfection.

**Reviewer #2:**

1. *The inhibitors have strong effects on the proliferation of the cells as do the knockouts. However, is this actually an effect specific to EBV infected B-cells. There are no controls for the general effect of these inhibitors on the proliferation of B-cells. They state that the desaturases are relatively ubiquitous in human cells. While they are increased in EBV infected cells, the effect of these inhibitors should be shown in uninfected cells.*

**Response:** We have addressed this comment in our response to comment #1 from reviewer #1 with the relevant data in reviewer **Figure 1 above and new Figure 2J-K and Supplementary Figure 4 in the manuscript)**.

As noted above: We thank the reviewer for this suggestion, and we agree that this would be valuable addition to our study. To our knowledge, the combined role of SCD1 and FADS2 had not yet been studied in uninfected, primary B cells. To address this, we stimulated PBMCs with either CpG or recombinant CD40L+IL-4 in the presence of cyclosporine A. Simultaneously, we treated stimulated PBMCs with SCD1i and/or FADS2i. We found that inhibition of either SCD1 or FADS2 decreased the number of CD19+ B cells that proliferated by day five in both stimulation conditions (**new Figure 2J-K and Supplementary Figure 4**). However, similar to LCLs, treatment with both SCD1i and FADS2i simultaneously further decreased the number of CD19+ B cells that proliferated in response to these mitogens. These data suggest that the EBV-driven SCD1/FADS2 upregulation and fatty acid desaturation help fulfill the metabolic requirements needed for B cells to divide.

2. *They state in the discussion line 550, “the observed cytotoxicity of palmitate upon dual SCD1 and FADS2 loss, suggests SCD1 and FADS2 prevent palmitate-induced lipotoxicity throughout infection” This actually is not borne out by the data. While the desaturases can prevent palmitate lipotoxicity, the data indicates that this does not occur in the cells they test as the inhibition of SCD1 and FADS2 leads to inhibition of proliferation not cell death. If they were protecting form lipotoxicity there would also be increased cell death. This is actually discussed later in the discussion when they point out that palmitate is not increased when they examine lipid species, further indicating that at least in the conditions tested in the manuscript, protecting from palmitate toxicity is not a critical mechanism for the desaturases. This should be properly discussed.*

**Response:** We thank the reviewer for pointing out this inconsistency in our discussion, and we agree with the point brought forward. The data from our study show that although SCD1 and FADS2 can prevent palmitate lipotoxicity, this is likely not their role during EBV-induced B cell transformation. We have clarified our statement from the original line 550 of the Discussion to more accurately represent this finding:

*“This observed expression of FASN throughout EBV-driven B cell immortalization, combined with the observed cytotoxicity of palmitate upon dual SCD1 and FADS2 loss, led us to hypothesize that SCD1 and FADS2 prevent palmitate-induced lipotoxicity throughout infection. However, we did not observe an increase in palmitate levels within the free fatty acid pool after SCD1+FADS2 inhibition, suggesting that the cell cycle arrest and reduction in growth-promoting metabolism associated with SCD1+FADS2 inhibition is not directly due to palmitate accumulation.”*

3. *The authors need to provide a stronger explanation for why there is a decreased saturated to unsaturated fatty acid profile upon inhibition of the desaturases as this is directly counter to what would be expected.*

**Response:** We have added a more in-depth discussion of our hypothesis that the increase in free PUFA abundance after desaturase inhibition might be due to lipolysis of complex lipids. We highlight a recent study showing that SCD1 inhibition in T cells results in an increase in non-esterified docosahexaenoic acid due to ATGL-driven lipolysis [8]. This hypothesis is therefore logical and warrants future investigation. Additionally, we would like to point out the possibility of excess palmitate not being detected because of esterification after biosynthesis. We profiled only free fatty acids because desaturase inhibition hypersensitized LCLs to free BSA-conjugated palmitate. Other studies that investigate the ability for FADS2 to compensate for a loss of SCD1 activity in other cellular contexts have also relied on only free fatty acid profiling [9]. However, because of the limited scope of free-fatty acid profiling, we may have missed accumulating levels of esterified palmitate in pools of other lipid species.

4. *Figure 1B is not explained clearly enough to understand the significance*

**Response:** We have added a more detailed explanation of **Figure 1B** in the results, including what we mean by “B-to-LCL transition” and why the upregulation of SCD1 and FADS2 led us to use Gene Set Enrichment Analysis to test whether the entire fatty acid desaturation gene set was differentially expressed between B cells and LCLs.

5. *Figure 3B and C: Is it possible that transfection of LCLs with multiple RNP complexes decreases the efficiency of knockout compared to one RNP which would give similar decreased results?*

**Response:** Transfecting LCLs with multiple RNP complexes does not, in and of itself, decrease transfection efficiency (**Figure 5 below** provides an example of targeting CD46 along with either CD226 or CD226+CD21). Additionally, even if transfection efficiency is lower with multiple guides, the % transfected remains stable over time if the targeted genes have no effect on cell proliferation. We observed a decrease in % transfected (i.e. %CD46-) over time after targeting CD46+SCD1+FADS2 together, suggesting that dual targeting of SCD1 and FADS2 slows or prevents cell division in LCLs. Targeting CD46 alone did not affect %CD46- over time. This decrease in % transfected over time is similarly observed in LCLs transfected with guides targeting CD46 and MDM2, which is a negative regulator of apoptosis, providing a proof of concept for this methodology.

**Figure 5: More RNP complexes does not limit transfection efficiency.** LCLs were transfected with Cas9-RNP complexes targeting CD46 and CD226, or CD46, CD226, and CD21. Transfection efficiencies were 61.3% and 62.8%, respectively.

6. *Figure 5A is presented as a percent of no inhibitor, but does Oleate have any effect on cell number when treated? Could these be shown as relative to no treatment at all to show the effects of palmitate and oleate on the untreated cells?*

**Response:** We thank the reviewer for the suggestion. We have updated **Figure 5A** in the manuscript to show values normalized only to the DMSO+BSA treatment (**Figure 6**), and we have included this updated figure in our results. Treatment with 100μM oleate-BSA or palmitate-BSA alone does not significantly affect LCL growth.

**Figure 6: Oleate alone does not affect the growth of LCLs.** LCLs treated with indicated desaturase inhibitors and different combinations of BSA-conjugated fatty acids. Relative cell number measured using CellTiter Glo (n=4).

7. *Panel 4G: Y-axis states relative cell number but looks to be actual cell number, not relative*

**Response:** In this panel, the label “relative cell number” refers to the Relative Luciferase Unit (RLU) measurement that one obtains when assessing cell number using CellTiter Glo. Although the values you are referring to are not normalized to a baseline or control (i.e. to DMSO or BSA), they are still relative because one RLU does not directly correspond to one cell and is a measurement of the amount of ATP in a sample. Instead, RLU values and cell numbers directly correlate with one another. As we indicate that relative cell numbers were determined using CellTiter Glo in each figure caption, we believe that the data are accurately presented.

**Reviewer #3:**

1. *It is appreciated that the authors choose their words carefully regarding dual SCD1 and FADS2 inhibitors. The work would be improved and conclusions strengthened if the authors formally tested if A939572 (SCD1i) and SC-26196 (FADS2i) are additive or synergistic (superadditive).*

**Response:** We thank the reviewer for this helpful suggestion. We were able to obtain IC50 values at 48 and 72hrs post treatment for SCD1i (48.5 and 40.9μM, respectively). However, we were unable to obtain IC50 values for FADS2i due to solubility constraints. Representative dose-response curves at 48hrs are shown in **Figure 7A-B below**. In lieu of an IC50 value for FADS2, we generated isobolograms using a [FADS2i] lower than its actual IC50. We used 50μM because this was the maximum dose we were able to treat LCLs with before the solubility limit was reached, resulting in a 10-15% growth inhibition. Even with this sub-IC50 concentration of FADS2i, isobolograms revealed that SCD1i and FADS2i are synergistic/superadditive with as low as 2μM FADS2i (**Figure 7C-E below**). These data are now presented as **Supplementary Figure 2** in the manuscript.

**Figure 7: Dose response data and isobolograms for SCD1i+FADS2i.** **A**) Dose-response data for SCD1i. Relative cell number measured using CellTiter Glo and normalized to DMSO. **B**) Dose-response data for FADS2i. Relative cell number measured using CellTiter Glo and normalized to DMSO. **C-D**) Isobologram at 48hrs and 72hrs, respectively, using IC50 for SCD1i on y axis and sub-IC50 (maximum soluble concentration) for FADS2i on x axis. Plotted points show IC50 values for SCD1i in the presence of that concentration of FADS2i. **E**) Drug combination indices (CI) at indicated dose of FADS2i. A CI value less than 1 indicates synergism.

2. *The study only examined some free fatty acids (FFA). Since they only looked at one, very narrow type of lipid, the authors should consider their usage of ‘lipidomics’. Since a full lipidomic analysis may yield additional or novel insight, it may be more appropriate to describe the current work as FFA analysis or FFA profiling.*

**Response:** We agree with the reviewer that our analysis is more accurately described as FFA profiling, and we have changed the terminology used within the manuscript.

3. *Figs. 1, 3: The FADS2 blots are faint/hard to interpret. Could the authors provide better images? Relatedly, if the authors state the number of unique peptides that are contributing to the levels of SCD1 and FADS2 in Fig. 1D then the readers will be able to more appropriately judge the data.*

**Response:** Three peptides specific to FADS2 and one peptide specific to SCD were detected in the mass spectrometry-based proteomics experiment shown in Figure 1D of the manuscript (dataset originally from Ersing, et al., 2017 [10]). We have also adjusted the contrast on the Western blot images and provided densitometry quantifications for the knockout (**Figure 8A-B below**, also added to manuscript as **Supplementary Figure 3**). FADS2 protein abundance was reduced by approximately 30% by Cas9-RNP targeting, which may reflect the short amount of time cells were allowed to grow post transfection before sorting/lysis (five days), growth arrest in the targeted cells, and/or low turnover of FADS2 proteins. We validated that the sgRNAs targeting FADS2 were on-target by sorting LCLs co-transfected with sgRNAs targeting CD46 and FADS2 and sequencing genomic DNA encompassing the FADS2 cut-sites in targeted and non-targeted cells. We then used Synthego’s Inference of CRISPR Edits tool to predict the editing efficiency of our transfection reaction. The model predicted that 98% of cells targeted with sgRNAs against FADS2 had indels in the FADS2 coding sequence (**Figure 8C-D below**, and in **manuscript within Supplementary Figure 5**), confirming the on-target nature of our FADS2 sgRNA.

**Figure 8: Targeting FADS2 with Cas9-RNP complexes does not fully deplete FADS2 levels, but sgRNAs are on-target.** **A**) Western blot showing abundance of FADS2, SCD1, and MAGOH (loading control) after targeting with Cas9-RNP complexes. **B**) Quantification of panel A, normalized to MAGOH and WT. **C**) Genomic FADS2 sequences in WT and FADS2-KO LCLs. Black lines indicate binding sites of FADS2 sgRNAs. **D**) Discordance of FADS2 genomic sequences between WT and CD46+FADS2 KO LCLs (left), and estimated percentage of each indel in mixture (right).

4. *Fig. 5A: It appears that 6 DMSO conditions were set to 100%. Why did the authors choose to do this instead of setting everything relative to DMSO 0µM palmitate / 0µM oleate?*

**Response:** This item was also addressed in our response to Reviewer 2 comment 6. The requested data are shown in reviewer **Figure 6 above**. We have updated **Figure 5A** in our manuscript to show values normalized only to the DMSO+BSA treatment. Treatment with 100μM oleate-BSA or palmitate-BSA alone does not significantly affect LCL growth.

5. Fig. 5D: Please remake this figure, it is impossible to read.

**Response:** We have made the font size of fatty acid labels larger and more legible.

6. *Fig. 5F: The desaturation reaction consumes oxygen. Does dual SCD1i + FADS2i treatment reduce the non-mitochondrial oxygen consumption rate?*

**Response:** We compared oxygen consumption rate (OCR) of LCLs treated with either DMSO or SCD1i+FADS2i, approximately ten minutes after inhibition of both mitochondrial ATP production with oligomycin and mitochondrial oxygen consumption with Rotenone/Antimycin A. Rotenone and Antimycin A are inhibitors of mitochondrial complex I and III, respectively [11] [12]. LCLs from two donors treated with SCD1i+FADS2i had significantly lower levels of non-mitochondrial oxygen consumption at the 48-hour treatment timepoint compared to DMSO-treated controls (**Figure 9A-B below**), which helps validate that the compounds are indeed inhibiting SCD1 and FADS2.

**Figure 9: SCD1+FADS2 inhibition decreases non-mitochondrial O^2^ consumption.** **A**) Oxygen consumption rate over time after SCD1+FADS2 inhibition. Red arrow indicates the timepoint after full electron transport chain inhibition where non-mitochondrial O^2^ consumption was measured. **B**) Comparison of non-mitochondrial O^2^ consumption rates between DMSO-treated and SCD1i+FADS2i-treated LCLs.

7. *Fig. 6A: Please consider changing the symbols or colors to enable the FADS2i data to be shown.*

**Response:** We have changed the size and layering order of the FADS2i data points, allowing them to be more visible over the SCD1i data. Because the IBL-1 cell line was derived from a single patient [13], we can expect less variation between biological replicates when compared to experiments in LCLs derived from multiple donors.

**REFERENCES**

1.	Sjögren P, Sierra-Johnson J, Gertow K, Rosell M, Vessby B, de Faire U, et al. Fatty acid desaturases in human adipose tissue: relationships between gene expression, desaturation indexes and insulin resistance. Diabetologia. 2008;51: 328–335. doi:10.1007/s00125-007-0876-9

2.	Ternes P, Franke S, Zähringer U, Sperling P, Heinz E. Identification and Characterization of a Sphingolipid Δ4-Desaturase Family*. J Biol Chem. 2002;277: 25512–25518. doi:10.1074/jbc.M202947200

3.	Davenport MG, Pagano JS. Expression of EBNA-1 mRNA Is Regulated by Cell Cycle during Epstein-Barr Virus Type I Latency. J Virol. 1999;73: 3154. doi:10.1128/jvi.73.4.3154-3161.1999

4.	Lorito N, Subbiani A, Smiriglia A, Bacci M, Bonechi F, Tronci L, et al. FADS1/2 control lipid metabolism and ferroptosis susceptibility in triple-negative breast cancer. EMBO Mol Med. 2024;16: 1533–1559. doi:10.1038/s44321-024-00090-6

5.	Yamane D, Hayashi Y, Matsumoto M, Nakanishi H, Imagawa H, Kohara M, et al. FADS2-dependent fatty acid desaturation dictates cellular sensitivity to ferroptosis and permissiveness for hepatitis C virus replication. Cell Chem Biol. 2022;29: 799-810.e4. doi:10.1016/j.chembiol.2021.07.022

6.	Kim JW, Lee J-Y, Oh M, Lee E-W. An integrated view of lipid metabolism in ferroptosis revisited via lipidomic analysis. Exp Mol Med. 2023;55: 1620–1631. doi:10.1038/s12276-023-01077-y

7.	Forte E, Luftig MA. MDM2-Dependent Inhibition of p53 Is Required for Epstein-Barr Virus B-Cell Growth Transformation and Infected-Cell Survival. J Virol. 2009;83: 2491–2499. doi:10.1128/JVI.01681-08

8.	Grajchen E, Loix M, Baeten P, Côrte-Real BF, Hamad I, Vanherle S, et al. Fatty acid desaturation by stearoyl-CoA desaturase-1 controls regulatory T cell differentiation and autoimmunity. Cell Mol Immunol. 2023;20: 666–679. doi:10.1038/s41423-023-01011-2

9.	Ikeda T, Katoh Y, Hino H, Seta D, Ogawa T, Iwata T, et al. FADS2 confers SCD1 inhibition resistance to cancer cells by modulating the ER stress response. Sci Rep. 2024;14: 13116. doi:10.1038/s41598-024-64043-2

10.	Ersing I, Nobre L, Wang LW, Soday L, Ma Y, Paulo JA, et al. A Temporal Proteomic Map of Epstein-Barr Virus Lytic Replication in B Cells. Cell Rep. 2017;19: 1479–1493. doi:10.1016/j.celrep.2017.04.062

11.	Heinz S, Freyberger A, Lawrenz B, Schladt L, Schmuck G, Ellinger-Ziegelbauer H. Mechanistic Investigations of the Mitochondrial Complex I Inhibitor Rotenone in the Context of Pharmacological and Safety Evaluation. Sci Rep. 2017;7: 1–13. doi:10.1038/srep45465

12.	Pham N-A, Robinson BH, Hedley DW. Simultaneous detection of mitochondrial respiratory chain activity and reactive oxygen in digitonin‐permeabilized cells using flow cytometry. 2000 [cited 18 Dec 2024]. Available: https://onlinelibrary.wiley.com/doi/10.1002/1097-0320(20001201)41:4<245::AID-CYTO2>3.0.CO;2-#

13.	Guasparri I, Bubman D, Cesarman E. EBV LMP2A affects LMP1-mediated NF-κB signaling and survival of lymphoma cells by regulating TRAF2 expression. Blood. 2008;111: 3813–3820. doi:10.1182/blood-2007-03-080309
